# Skyrmion Bubbles by Design in a Centrosymmetric Kagome Magnet

**DOI:** 10.1002/advs.78269

**Published:** 2026-10-20

**Authors:** Mohamed El Gazzah, Fehmi Sami Yasin, Sk Jamaluddin, Hari Bhandari, Resham Babu Regmi, Xiuzhen Yu, Nirmal J. Ghimire

**Affiliations:** ^1^ Department of Physics and Astronomy University of Notre Dame Notre Dame Indiana USA; ^2^ Stavropoulos Center For Complex Quantum Matter University of Notre Dame Notre Dame Indiana USA; ^3^ Center for Nanophase Materials Sciences Oak Ridge National Laboratory Oak Ridge Tennessee USA; ^4^ RIKEN Center for Emergent Matter Science Wako Japan; ^5^ Department of Physics and Astronomy Rice University Houston Texas USA; ^6^ Institute of Science Tokyo Tokyo Japan

**Keywords:** condensed matter physics, cryogenic Lorentz DPC‐STEM, Lorentz transmission electron microscopy, magnetocrystalline anisotropy, nanoscopic scale, skyrmion, spintronics

## Abstract

Topologically protected nanoscale spin textures, such as magnetic skyrmions, have attracted significant interest for spintronics applications. While skyrmions in noncentrosymmetric materials are known to be stabilized by the Dzyaloshinskii‐Moriya interaction (DMI), their deliberate design in centrosymmetric materials remains a challenge. This difficulty largely stems from the complexity of controlling magnetocrystalline anisotropy–a critical factor in the absence of DMI. Here, we demonstrate the chemical tuning of magnetocrystalline anisotropy in the centrosymmetric Kagome magnet ${\mathrm{TmMn}}_{6}{\mathrm{Sn}}_{6}$. The resulting compound exhibits a spin reorientation transition accompanied by an emergent skyrmion bubble lattice, confirmed by cryogenic Lorentz transmission electron microscopy and cryogenic Lorentz differential phase contrast scanning transmission electron microscopy. Our findings overcome a key materials design challenge and open possibilities for deliberate design of skyrmionic textures in centrosymmetric systems.

## Graphical abstract

Chemical tuning of magnetocrystalline anisotropy in the centrosymmetric kagome magnet TmMn_6_Sn_6_ is shown to drive a spin reorientation transition via an intermediate magnetic fan state and to stabilize an emergent skyrmion bubble lattice. Four‐dimensional cryogenic Lorentz scanning transmission electron microscopy confirms the nanoscale texture, demonstrating a practical route to engineer skyrmionic states in centrosymmetric materials.

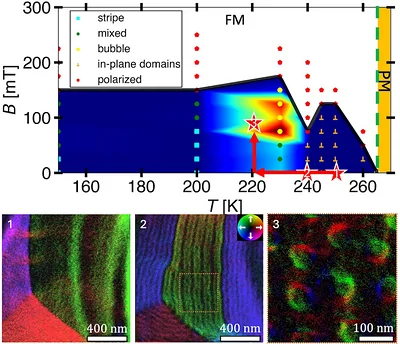

## 1 Introduction

The identification, understanding, and manipulation of novel magnetic textures is essential for the characterization of new quantum materials which may compose future spin‐based electronic devices [1, 2, 3]. Among these textures, magnetic skyrmions, vortex‐like spin textures with integer topological charge S, have garnered particular attention due to their intriguing physical properties and technological potential [4, 5, 6, 7]. Skyrmions were first discovered in 2009 in the noncentrosymmetric helimagnet MnSi [4], where their stabilization was governed by the antisymmetric Dzyaloshinskii–Moriya interaction (DMI), a consequence of broken inversion symmetry in the crystal lattice [8, 9]. In such systems, a delicate balance between DMI, symmetric exchange interactions, and magnetocrystalline anisotropy facilitates the formation of these topologically protected spin textures. The excitement surrounding skyrmions intensified in 2010 when it was shown that they could be manipulated using electric current densities five orders of magnitude smaller than those required for conventional ferromagnetic metals and semiconductors [10] ‐ highlighting their promise for low‐power spintronic applications [11, 12, 13, 14].

Since then, skyrmions have been identified in a wide range of bulk materials and artificially layered heterostructures [15, 16, 17]. In the latter, interfacial DMI emerges from symmetry breaking at material interfaces, mirroring the stabilization mechanism seen in noncentrosymmetric helimagnets. Consequently, early research efforts focused mainly on DMI‐driven skyrmion formation [18, 19, 20], under the assumption that DMI was a necessary ingredient. This assumption has been fundamentally challenged by recent discoveries of skyrmions in centrosymmetric material systems that lack DMI entirely [21, 22, 23, 24, 25]. In these cases, skyrmion formation arises from a more complex interplay of magnetic and geometric frustration, higher‐order exchange interactions, dipolar coupling, and magnetocrystalline anisotropy. These findings have expanded the landscape of materials that can host skyrmions and raise important questions about the deeper mechanisms that govern their stabilization [26, 27, 28, 29].

Despite these advances, a unifying characteristic across most known skyrmion‐hosting systems is that their emergence has been passive: they have been stabilized solely by the already present interactions within the material. This raises a pivotal question: Can we move beyond discovery and toward the active engineering of skyrmions on demand? Recent work demonstrating the ability to tune the magnetic properties of thin film magnets via focused ion beam (FIB)‐induced ion implantation suggests that such control is possible [30, 31]. Achieving this control in bulk single crystals would be a remarkable step in topological magnetism, enabling deliberate design and manipulation of skyrmionic textures for future spintronics applications. This would not only allow for control over the size and density of skyrmions, considered important for technological applications [32], but also open avenues for coupling them with electronic topological states, potentially leading to more exotic phenomena [3, 33, 34, 35, 36, 37]. These possibilities echo the theoretical predictions of real‐ and momentum‐space topology interplay, such as those proposed in the A‐phase of superfluid helium‐3 [38, 39] and more recently discussed theoretically in condensed matter systems [33, 34, 36, 40]. However, a major challenge in the proposed strategies for engineering skyrmions in centrosymmetric systems [41] lies in the control of magnetocrystalline anisotropy [42]. As a result, previous efforts have primarily focused on materials incorporating weakly anisotropic rare‐earth elements such as Gd [43, 44, 45] and Eu [46, 47, 48, 49].

In this article, we demonstrate a controlled design of skyrmion bubbles and their manipulation from Type‐I (integer topological charge, $S=\frac{1}{4\pi }\int \int m\cdot \left(\frac{\partial m}{\partial x}\times \frac{\partial m}{\partial y}\right)\mathrm{dx}\;\mathrm{dy}=-1$, where $m=\frac{M}{\left|M\right|}$ is the normalized magnetization) to Type‐II (non‐topological, S=0) shown in Figure 1a, b, in the centrosymmetric Kagome lattice compound ${\mathrm{TmMn}}_{6}{\mathrm{Sn}}_{6}$. To stabilize these spin textures, we engineer the magnetocrystalline anisotropy through targeted chemical substitution, similar to the recent work done in the schreibersite ${\left(Fe,Ni\right)}_{3}P$ with $S_{4}$ symmetry [42]. We use real‐space imaging via cryogenic Lorentz transmission electron microscopy (LTEM) and cryogenic Lorentz differential phase contrast scanning tranmission electron microscopy (LDPC‐STEM) to show that the stabilization of a skyrmion bubble lattice occurs immediately below the engineered spin reorientation transition, where competing anisotropies from the rare‐earth and transition‐metal sublattices are maximal. Our results reveal a skyrmionic bubble lattice phase that persists across a wide temperature and field window. Furthermore, we observe spontaneous skyrmion bubble helicity switching and the coexistence of opposite helicities‐features that highlight the complex and highly sensitive energy landscape of these spin textures as well as the apparent degeneracy of both bubble helicities. Our findings offer a new pathway for centrosymmetric materials: by harnessing competing anisotropies through chemical substitution, one can tune the system to the optimal conditions of realizing a spin reorientation transition. Crucially, the magnetic anisotropy can be tuned via chemical substitution, enabling the deliberate induction of a spin reorientation transition resulting in the stabilization of topological spin textures in centrosymmetric systems by design.

**FIGURE 1:**
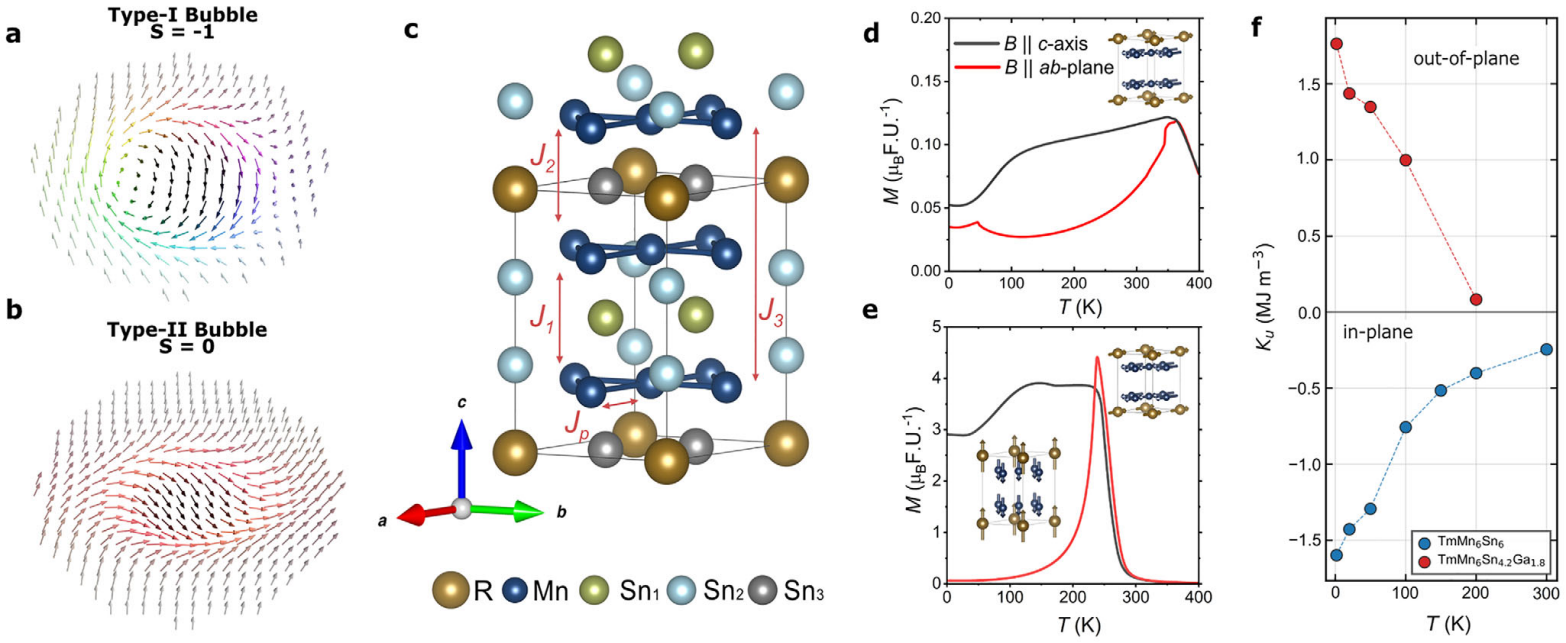
Structural, magnetic, and topological characteristics of ${\mathrm{TmMn}}_{6}{\mathrm{Sn}}_{6}$. (a‐,) Schematics of (a) a Bloch Type‐I magnetic skyrmion bubble and (b) a Type‐II non‐topological magnetic bubble. (c) Hexagonal P6/mmm (191) crystal structure of ${\mathrm{TmMn}}_{6}{\mathrm{Sn}}_{6}$ where the exchange interactions between the Kagome Mn layers are denoted $J_{1}$, $J_{2}$, and $J_{3}$, and the exchange interaction between Mn moments within the layer is denoted $J_{P}$. (d) Field‐cooled temperature‐dependent magnetization of the parent compound ${\mathrm{TmMn}}_{6}{\mathrm{Sn}}_{6}$ under an applied field of 0.1 T along both the ab‐plane (red curve) and c‐axis (black curve). (e) Field‐cooled temperature‐dependent magnetization of Ga‐doped ${\mathrm{TmMn}}_{6}{\mathrm{Sn}}_{6}$, ${\mathrm{TmMn}}_{6}{\mathrm{Sn}}_{4.2}{\mathrm{Ga}}_{1.8}$, under the same conditions with a clear spin reorientation appearing around 243 K. (f) Evolution of anisotropy obtained from the magnetization measurements as a function of T, for both ${\mathrm{TmMn}}_{6}{\mathrm{Sn}}_{6}$ and ${\mathrm{TmMn}}_{6}{\mathrm{Sn}}_{4.2}{\mathrm{Ga}}_{1.8}$ indicated by blue and red circular markers, respectively.

## 2 Spin Reorientation Within TmMn_6_Sn_6_

${\mathrm{TmMn}}_{6}{\mathrm{Sn}}_{6}$ belongs to the broader family of hexagonal ${\mathrm{RMn}}_{6}{\mathrm{Sn}}_{6}$ compounds, which have recently garnered significant attention for their complex and tunable magnetic behavior. This richness arises from a delicate interplay of competing interlayer exchange interactions–$J_{1}$, $J_{2}$, and $J_{3}$–as illustrated in Figure 1c [50, 51, 52]. Within this family, ${\mathrm{YMn}}_{6}{\mathrm{Sn}}_{6}$ has been proposed to host a thermally stabilized dynamical skyrmion phase, believed to underlie its observed topological Hall response [50]. Similarly, in ${\mathrm{TbMn}}_{6}{\mathrm{Sn}}_{6}$, a biskyrmion lattice has been reported to emerge exclusively near a spontaneous spin reorientation transition [53], suggesting that such a reorientation may be key to stabilizing topological spin textures.

Recent work has shown that the spin reorientation in ${\mathrm{TbMn}}_{6}{\mathrm{Sn}}_{6}$ originates from competition between the magnetic anisotropies of the Mn and Tb sublattices, modulated by thermally driven spin fluctuations consistent with Mermin–Wagner physics [51]. Both sublattices order magnetically at about 420 K, but at elevated temperatures, the stronger in‐plane anisotropy of Mn sub‐lattice dominates. Below 310 K, however, the out‐of‐plane anisotropy of the Tb moments prevails, leading to a spontaneous spin reorientation of both Mn and Tb spins along the c‐axis. In contrast, ${\mathrm{TmMn}}_{6}{\mathrm{Sn}}_{6}$ exhibits robust in‐plane anisotropy over the entire temperature range [54, 55], as evidenced by the temperature‐dependent magnetization data (Figure 1d). These data show that the magnetic response along the c‐axis consistently exceeds that within the ab‐plane, a direct consequence of the in‐plane AFM ordering suppressing the net ab‐plane response relative to the c‐axis response. Remarkably, substitution of Ga into ${\mathrm{TmMn}}_{6}{\mathrm{Sn}}_{6}$ induces a clear spin reorientation from in‐plane to out‐of‐plane below 243 K, as indicated by the crossover of the in‐plane and out‐of‐plane magnetizations in Figure 1e. Ga preferentially substitutes at the Sn3 (2c) site within the Tm‐containing layer, modifying the Tm crystal‐field environment and enhancing the Tm easy‐axis on‐site anisotropy. Because the Tm moments occupy quasi‐two‐dimensional layers, their anisotropy is strongly suppressed by thermal fluctuations, while the Mn sublattice exhibits weaker thermal fluctuations because of its strong in‐plane exchange. Upon cooling, Tm easy‐axis anisotropy increases as thermal fluctuations are reduced and eventually exceeds the in‐plane anisotropy of the Mn sublattice, driving spin reorientation toward the c‐axis.

The uniaxial anisotropy constant $K_{u}$, estimated from magnetization measurements (see Magnetization and Anisotropy Calculation Section of Supporting Information), is plotted for both compounds in Figure 1f. The parent compound ${\mathrm{TmMn}}_{6}{\mathrm{Sn}}_{6}$ exhibits in‐plane anisotropy throughout the entire measured temperature range, consistent with the susceptibility measurements discussed above, whereas ${\mathrm{TmMn}}_{6}{\mathrm{Sn}}_{4.2}{\mathrm{Ga}}_{1.8}$ exhibits out‐of‐plane anisotropy. In both compounds, the magnitude of the anisotropy increases with decreasing temperature. Most notably, Ga substitution drives a crossover from in‐plane anisotropy in ${\mathrm{TmMn}}_{6}{\mathrm{Sn}}_{6}$ to out‐of‐plane anisotropy in ${\mathrm{TmMn}}_{6}{\mathrm{Sn}}_{4.2}{\mathrm{Ga}}_{1.8}$, as discussed in further detail in [56]. Furthermore, the ordering temperature of ${\mathrm{TmMn}}_{6}{\mathrm{Sn}}_{4.2}{\mathrm{Ga}}_{1.8}$ drops roughly 20% with respect to the parent compound, and using a mean‐field approximation, we find this leads to an 18% decrease in exchange stiffness energy, suggesting Ga substitution favors formation of less rigid domains (details can be found in Supporting Information). To probe the resulting magnetic textures, we employed high‐resolution magnetic domain imaging of Ga‐doped ${\mathrm{TmMn}}_{6}{\mathrm{Sn}}_{6}$ using cryogenic LTEM and LDPC‐STEM.

LTEM micrographs of a c‐axis wedge plate of ${\mathrm{TmMn}}_{6}{\mathrm{Sn}}_{4.2}{\mathrm{Ga}}_{1.8}$, with thicknesses of 91 nm in the darker region and 43 nm in the lighter region, are shown across temperatures from 260 to 200 K in Figure 2a–d. At 260 K, the thicker region reveals well‐defined in‐plane domains, identified through the existence of a dark‐intensity $180^{\circ }$ domain wall near the center of the lamella which branches to form two $90^{\circ }$ domain walls near the bottom edge of the LTEM micrograph. On the other hand, the thinner region shows no visible magnetic domain walls, indicating the presence of either a single magnetic domain or a paramagnetic state. Above the spin reorientation temperature $T_{\text{SR}}$ (at 245 K), in‐plane domains appear prominently in both thick and thin regions, confirming strong in‐plane anisotropy. At 240 K, the thicker region transitions to predominantly striped domains, indicative of a shift to out‐of‐plane anisotropy, while the thinner region retains in‐plane magnetic domains. Further cooling to 200 K results in both regions transitioning from in‐plane to out‐of‐plane anisotropy along the c‐axis, producing complex maze‐like striped domains across the entire lamella shown in Figure 2d.

**FIGURE 2:**
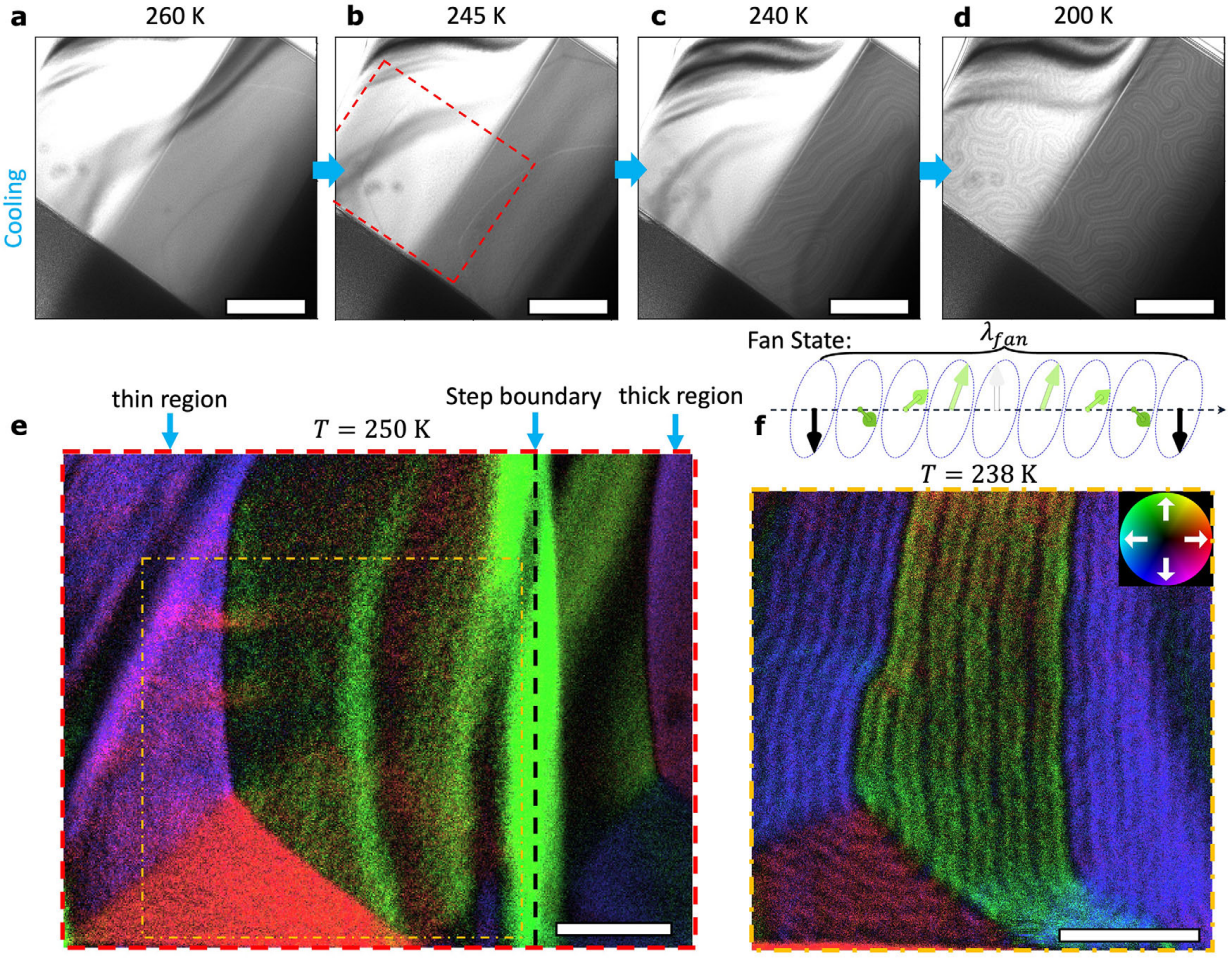
Real‐space evolution of magnetic textures across the spin reorientation transition. (a–d): Selected Lorentz transmission electron micrographs (LTEM) of magnetic textures in ${\mathrm{TmMn}}_{6}{\mathrm{Sn}}_{4.2}{\mathrm{Ga}}_{1.8}$ during zero‐field cooling from (a) 260 K to (b) 245 K, (c) 240 K, and (d) 200 K. Scalebars, 1μm.(e) Lorentz differential phase contrast scanning transmission electron microscopy (LDPC‐STEM) image of the selected region indicated by the dashed red rectangle outlined in (b) taken at 250 K and zero field, revealing ferromagnetic in‐plane domains. The black dashed line indicates the step‐like increase in thickness separating the thin region on the left‐hand side from the thick region on the right‐hand side. (f) LDPC‐STEM image of the selected area in the thin region indicated by the yellow dot‐dashed rectangle outlined in (e) at the spin reorientation temperature 238 K, revealing a magnetic fan state (shown schematically at the top of panel (f) where the magnetization periodically twists from out‐of‐plane to in‐plane to out‐of‐plane before reversing direction and sweeping a ‘fan’ shape (perfect semicircle) over the period $\lambda_{\text{fan}}$) which acts as the intermediate state between in‐ and out‐of‐plane spin states. Scalebars, 0.4μm.

To investigate the spin structure around the spin reorientation temperature, we performed LDPC‐STEM. The in‐plane magnetic induction maps at T=250K and T=238K are shown in Figure 2e–f. At 250 K, we find in‐plane magnetic domains in both regions of the lamella separated by either $180^{\circ }$ (e.g., between purple and green domains) or $90^{\circ }$ (e.g., between purple/green and red/blue domains) domain walls. At 238 K in the thin region indicated by the yellow dot‐dashed box in Figure 2e, a periodic structure emerges that LDPC‐STEM reveals in Figure 2f to be a fan state. The spins in this fan state continuously trace a fan‐like shape (perfect semicircle spanning $180^{\circ }$), twisting from in‐plane to out‐of‐plane across the period ($\lambda_{\text{fan}}$) of the texture, as shown in the schematic above Figure 2f. These magnetic fan states have modulation q‐vectors that align perpendicular to either the edge (for the red/blue domains) or the $180^{\circ }$ domain walls. As the temperature decreases further and the uniaxial anisotropy strengthens, the fan‐like spin modulations reorient smoothly into the labyrinth domains separated by $180^{\circ }$ domain walls shown in Figure 2d.

## 3 Emergence of Skyrmionic Bubbles

Below the spin reorientation temperature, the magnetic textures resemble those of prototypical magnets with uniaxial anisotropy. As such, the application of an external magnetic field parallel to the c‐axis drives the formation of Type‐I and Type‐II bubbles, illustrated in the magnetic field sweep in Figure 3a‐d at 240 K. At zero field (Figure 3a), the thicker region exhibits maze‐like magnetic domains with narrow domain walls. Upon application of a B=100mT magnetic field, the stripe domains widen in the regions with magnetization aligned parallel to the applied field and shrink in the anti‐parallel domains as shown in Figure 3b. At B=150mT, skyrmion bubbles stabilize and form a loose triangular lattice (Figure 3c). At B=200mT, the lamella saturates with spins polarized along the applied magnetic field (Figure 3d). Similar behavior is seen in the thinner region (Figure S4). In‐plane magnetic induction maps of Type‐I and Type‐II Bloch skyrmion bubbles are shown in Figure 3e, f, respectively. These two bubble types are observed in both the thick and thin regions, and so is their reversible transformation (between Type‐I and Type‐II bubbles) via the application of an oblique magnetic field (sample tilt), similar to prior work [57].

**FIGURE 3:**
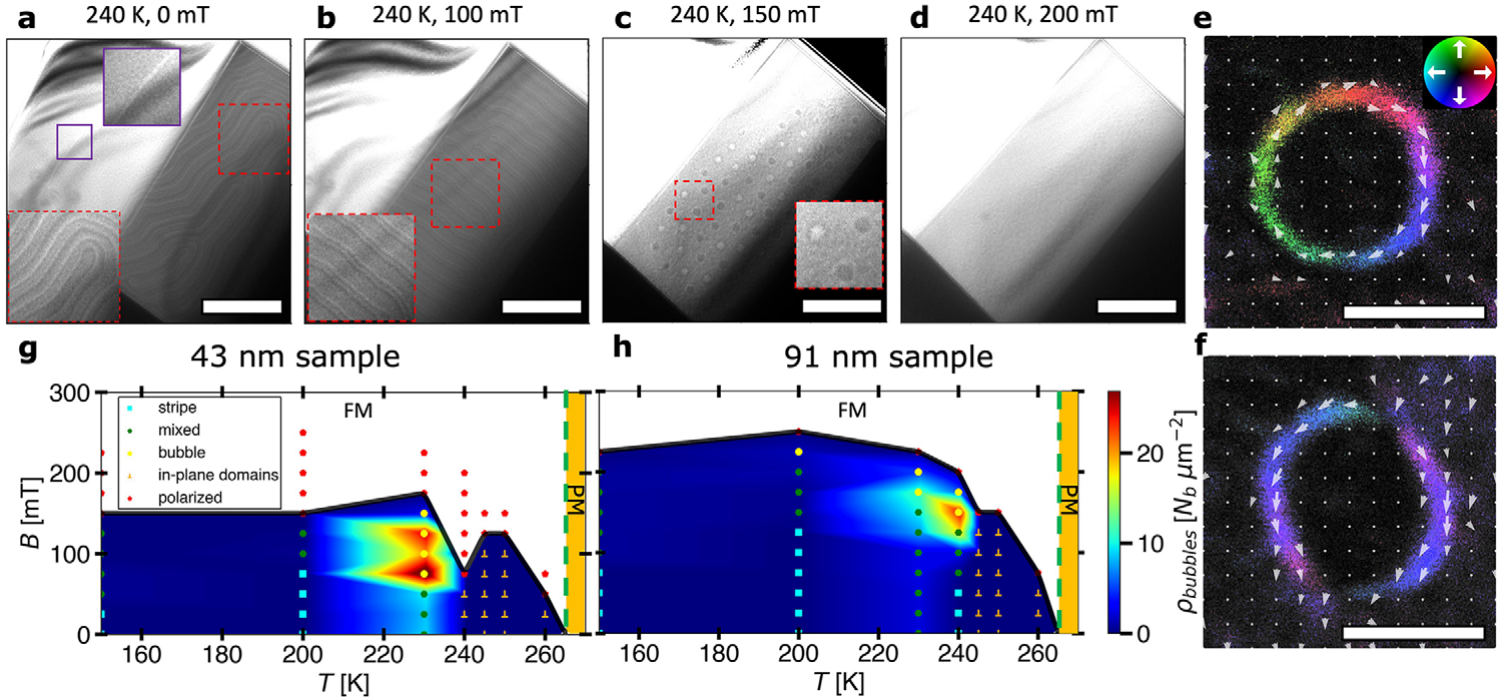
Thickness dependent magnetic‐field–temperature magnetic state diagrams of ${\mathrm{TmMn}}_{6}{\mathrm{Sn}}_{4.2}{\mathrm{Ga}}_{1.8}$. (a–d) Selected LTEM micrographs of a thin and thick region in the ${\mathrm{TmMn}}_{6}{\mathrm{Sn}}_{4.2}{\mathrm{Ga}}_{1.8}$ lamella depicting the magnetic states at T=240 K under external magnetic fields of (a) $B_{\text{ext}}=0$ mT, (b) $B_{\text{ext}}=100$ mT, (c) $B_{\text{ext}}=150$ mT, and (d) $B_{\text{ext}}=200$ mT. Scalebars, 1μm. The magnetic states evolve from (a) out‐of‐plane domains with maze‐like modulations and $180^{\circ }$ domain walls at zero field to a (b) single q‐vector stripe state at B=100mT, likely due to the presence of a slight sample tilt and therefore a non‐zero in‐plane component of the magnetic field relative to the [001] crystal axis. Skyrmionic bubbles emerge at (c) B=150mT and the spins polarize into a ferromagnetic state above B=200mT. (e, f) In‐plane magnetic induction maps of a e Type‐I skyrmion bubble and a f Type‐II non‐topological bubble measured using LDPC‐STEM. Scalebars, 0.1μm.(g,h) The temperature T[K] versus applied magnetic field B[mT] magnetic state diagrams of the (g) 43nm thin and (h) 91nm thick regions of the thin plate. The magnetic bubble density $\rho_{\mathrm{bubbles}}$ is plotted in color, and the uniaxial anisotropic stripe domains, mixed stripe and magnetic bubbles, magnetic bubble lattice, in‐plane domains, and polarized states are indicated by cyan squares, green hexagons, yellow circles, orange ‘⊥,’ and red pentagons, respectively. The curie temperature is indicated by a green dashed vertical line and the ferromagnetic (polarized) and paramagnetic states are labeled as ‘FM’ and ‘PM’ respectively. Note that the FM state for temperatures above $T_{\text{SR}}$ indicates magnetic states where no in‐plane domain walls were observed. Because the magnetocrystalline anisotropy is in‐plane at these temperatures, the magnetic field required to polarize the spins along the *c*‐axis is likely very high and it is possible that the spins in these states contain an in‐plane component.

The magnetic state diagrams for the d=43nm and d=91nm thick regions are shown in Figure 3g, h. The spins order from a paramagnetic state labeled ‘PM’ in the yellow shaded regions on the right‐hand‐side of the green dashed line indicating the Curie temperature, to in‐plane domains (indicated by orange ⊥ markers), which become polarized (marked by red pentagons) under sufficiently high applied magnetic fields. This behavior is consistent in both the thick and thin regions, although a higher magnetic field is required to polarize the larger volume of spins in the thicker sample. At the spin reorientation temperature and zero field, a fan state was observed as discussed in Figure 2. Below this temperature, we observe a range of magnetic structures, including striped domains, bubbles, and mixed states consisting of bubbles and striped domains marked in Figure 3g, h as cyan squares, yellow circles, and green hexagons. Striped domains emerge at lower magnetic fields and transition into a mixed state with increasing field strength before fully transforming into bubbles. Further increases in the magnetic field lead to saturation of the thin plate into a ferromagnetic state labeled as ‘FM’ in Figure 3g, h.

In the 43‐nm region, a high density of magnetic bubbles, marked by yellow dots, is observed at 230 K at fields ranging from 60 to 140 mT. In contrast, the 91‐nm region exhibits a lower bubble density, forming at higher fields over a broader temperature range: at 240 K with 180 mT, at 230 K with 180–200 mT, and at 200 K with 220 mT. This behavior qualitatively follows what we would expect from Kittel's law [58], which states that the characteristic domain size D scales with the square root of the thickness due to dipolar interactions. In that work, the total energy of a thin film magnet with uniaxial anisotropy per unit area is derived to be $F=\sigma_{w}\left[2\sqrt{2}+\left(d-D\right)/D\right]+\varepsilon_{a}D/2$, where $\sigma_{w}$ is the surface energy density of the Bloch wall, $\varepsilon_{a}$ is the anisotropy energy density, and d is the film thickness. The energy is minimum when $D={\left(2\sigma_{w}d/\varepsilon_{a}\right)}^{1/2}$. In our case, we determined that the domain sizes are $D_{43\;\mathrm{nm}}=85\pm 17\;\mathrm{nm}$ and $D_{91\;\mathrm{nm}}=124\pm 12\;\mathrm{nm}$, respectively using the ground magnetic states after zero field cooling to T=150K as shown in Figure S6. This results in a scaling factor of $D_{91\;\mathrm{nm}}/D_{43\;\mathrm{nm}}=1.46$, which matches the expected $\sqrt{d_{thick}/d_{thin}}=\sqrt{91/43}=1.45$ dependence, providing quantitative evidence that dipolar interactions play a dominant role in setting the bubble size. In order to compare the two $\rho_{\mathrm{bubbles}}$ directly, we rescaled the magnetic bubble density in the thicker region (Figure 3h) by D(d=91nm)/D(d=43nm)∝91nm/43nm=2.12 [58].

The thickness‐dependent behavior highlights the critical interplay between dipolar interactions and anisotropy in stabilizing topological magnetic bubbles [59]. In thinner lamellae, the relative balance among domain‐wall, anisotropy, and magnetostatic energies changes, favoring smaller characteristic domain sizes and, under suitable fields, a higher density of magnetic bubbles. However, uniaxial anisotropy plays an equally crucial role, as it favors out‐of‐plane spin alignment, which is necessary for stabilizing these bubbles. To this extent, a quality factor Q can be defined as the ratio of anisotropy energy and the dipolar interaction: $Q=\frac{2K_{u}}{\mu_{0}M_{s}^{2}}$. As shown in Figure 1, $K_{u}$ increases from zero at the spin reorientation temperature for decreasing temperatures, leading to increasing Q. Interestingly, the largest skyrmion density occurs in these films in the soft uniaxially anisotropic regime 0<Q<1 and decreases to $\rho_{\mathrm{bubbles}}<1\;N_{b}\;\mu m^{-2}$ below T=100K (Q=1.69, see Table S2). This relationship is particularly evident in systems with reduced thickness, where the enhanced dipolar interactions, combined with sufficient uniaxial anisotropy, allow for the emergence of topological textures [59, 60, 61].

As discussed, the magnetic induction maps reveal two distinct magnetic bubbles stabilized in ${\mathrm{TmMn}}_{6}{\mathrm{Sn}}_{4.2}{\mathrm{Ga}}_{1.8}$, each with unique topological and magnetic characteristics. Type‐I bubbles (depicted in Figure 1a and shown in Figure 3e), are characterized by a circular domain surrounded by a Bloch‐type domain wall in which the in‐plane magnetization winds by $360^{\circ }$ around the bubble perimeter. The spin configuration in Type‐I bubbles wraps the unit sphere once, making them topologically non‐trivial and akin to skyrmions, imparting stability against thermal fluctuations and external perturbations. Such topological protection arises because the spins cannot be continuously deformed to a trivial state without breaking the continuity of the magnetization, making Type‐I bubbles particularly robust in varied magnetic environments. Typically, the topological Hall effect (THE) arises from the real‐space Berry curvature induced by the emergent magnetic field of nontrivial spin texture such as skyrmions. However, the present case deviates from that expectation. We suspect this is due to the relatively large size of the skyrmion bubbles (≈100nm), which reduces their emergent magnetic field that contributes to the topological Hall effect. Furthermore, the skyrmion bubble lattice is observed only in very thin samples (d≈50nm), which poses significant challenges for reliably measuring the Hall effect in such ultrathin specimens.

On the other hand, Type‐II bubbles (illustrated in Figure 1b and imaged in Figure 3f) exhibit a non‐topological structure. Here, the spins do not complete a full rotation around the perimeter; instead, they align symmetrically on opposite sides, converging at the Bloch lines, which are Néel‐like interfaces separating the two Bloch‐type domain wall segments. This configuration results in a state topologically equivalent to a striped domain or a polarized domain. In agreement with the literature, we find that Type‐II bubbles are stabilized under the application of small in‐plane magnetic fields, essential for balancing the competing magnetic anisotropies and dipolar interactions. This necessity for an oblique field confirms that Type‐II bubbles are more sensitive to external field orientation and magnitude than their topological counterparts.

The distinct magnetic properties of Type‐I and Type‐II bubbles have significant implications. The topological stability of Type‐I bubbles makes them promising candidates for information storage and manipulation in spintronics, where resilience to disturbances is crucial. In contrast, Type‐II bubbles, with their sensitivity to in‐plane fields and anisotropies, could be utilized in applications requiring rapid switching or modulation of magnetic states [9, 62, 63]. The ability to stabilize both through careful control of doping and external magnetic fields within the ${\mathrm{TmMn}}_{6}{\mathrm{Sn}}_{4.2}{\mathrm{Ga}}_{1.8}$ system showcases the versatility of the ${\mathrm{RMn}}_{6}{\mathrm{Sn}}_{6}$ family. This tunable magnetic behavior provides a powerful framework for engineering diverse magnetic textures and exploring new functional properties in quantum materials.

## 4 Thermally Driven Dynamic Bubble Helicity Flipping

During the real‐space microscopy experiments, we found clear signatures of helicity switching in Type‐I magnetic bubbles. Given the inherent topological protection of these bubbles, the helicity switching under applied field during LTEM imaging is particularly intriguing. While the topological nature of Type‐I bubbles makes them robust against collapse into a topologically trivial state, their helicity, or the chirality of the domain wall, is susceptible to dynamic switching between left‐handed and right‐handed configurations, while preserving integer topological charge, as both configurations are energetically equivalent due to the centrosymmetry of the underlying atomic lattice [64]. To selectively control the bubble chirality, the symmetry must be broken by applying, for example, an in‐plane electric field as was recently demonstrated in the 2D van der Waals magnet ${\mathrm{Cr}}_{2}{\mathrm{Ge}}_{2}{\mathrm{Te}}_{6}$ [65].

To quantify this phenomenon in ${\mathrm{TmMn}}_{6}{\mathrm{Sn}}_{4.2}{\mathrm{Ga}}_{1.8}$, we tracked the helicity switching rate of several bubbles across the thin region of the FIB‐fabricated lamella which has a wedge‐like thickness dependence. A defocused LTEM micrograph is shown in Figure 4a with circular markers of varying colors (thickness increases from cooler to warmer colors) drawn around the skyrmion bubbles selected for analysis. The thickness map of the lamella, measured using electron energy loss spectroscopy (EELS), is presented in Figure S4 [66]. Figure 4b shows step‐like changes in the integrated intensity within the bubbles' central core region, representing discrete helicity switching events because the vortex‐like in‐plane magnetic field focuses the electrons radially outward or inward depending on helicity. The rate of helicity switching $\Delta N_{\text{switch}}$ is plotted as a function of average sample thickness at the bubbles' locations in Figure 4c. The strong thickness dependence suggests that the energy barrier for a defect‐mediated helicity‐reversal pathway decreases as the lamella thickness is reduced.

**FIGURE 4:**
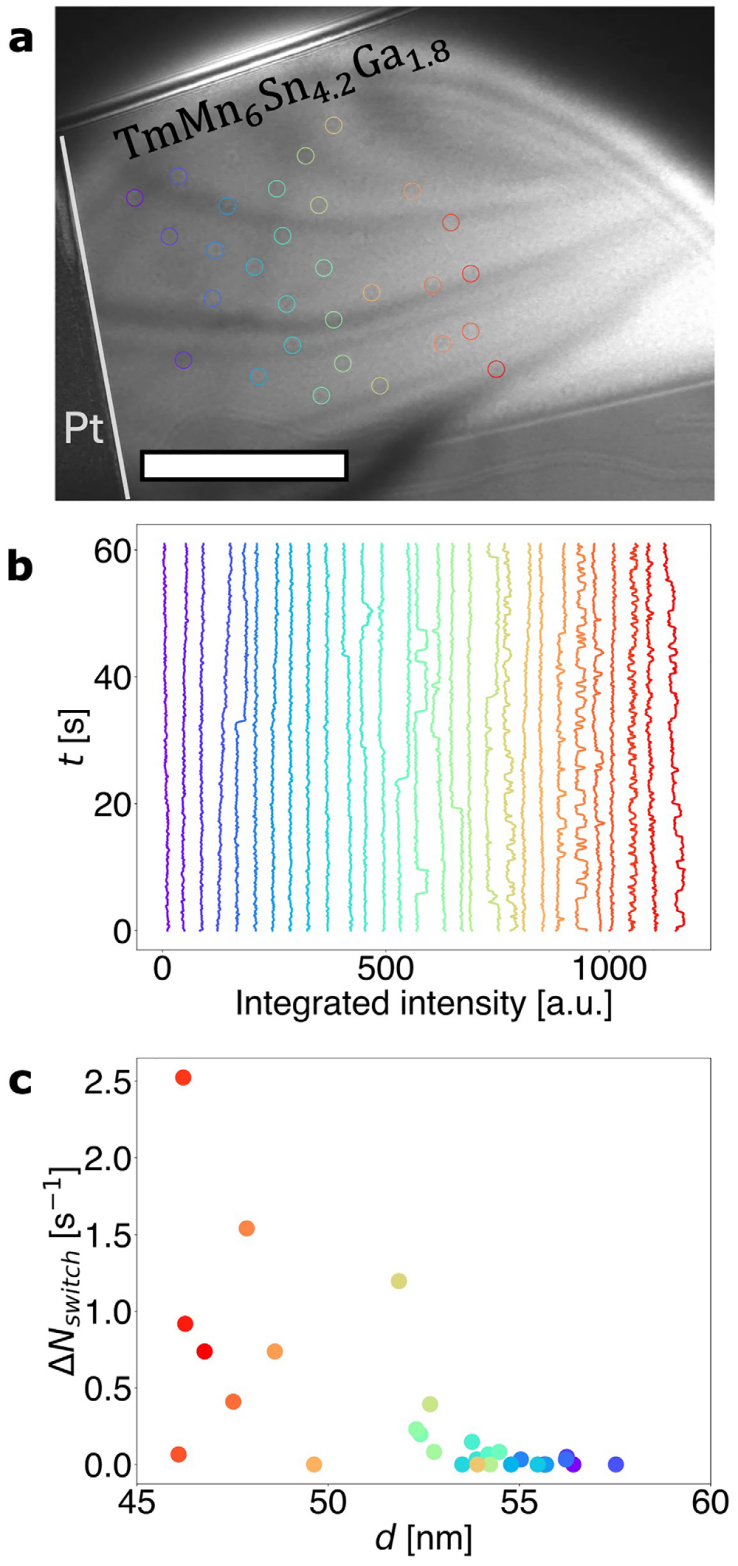
Thickness‐dependent helicity switching of magnetic bubbles. (a‐c): Helicity switching of magnetic bubbles in ${\mathrm{TmMn}}_{6}{\mathrm{Sn}}_{4.2}{\mathrm{Ga}}_{1.8}$ at $B_{\text{ext}}=125$ mT and T=210 K. (a) LTEM micrograph showing the thermodynamically stable magnetic bubble lattice state, with bubbles selected for helicity switching analysis marked by solid line circles in different colors. Scalebar is 1μm. (b) Time evolution of the integrated intensity within the selected bubbles, where the step‐like changes in intensity indicate helicity switching events. (c) The rate of helicity switching $\Delta N_{\text{switch}}$ as a function of thickness averaged over the bubbles' in‐plane area, illustrating the impact of lamella thickness on helicity switching rates.

One possible explanation for this behavior is the increased shape anisotropy in thinner regions, enhancing the role of the surface demagnetization field. Correspondingly, increased Néel‐type spin twisting near the sample surfaces may reduce the energy barrier to create and annihilate magnetic defects such as Bloch lines and Bloch points, similar to the dipolar interaction‐driven creation of Bloch points in electron‐transparent thin plates [67], and resulting in an increase in thermally‐activated helicity switching events which could lead to a motional narrowing effect on the spin dynamics. This observation aligns with our findings that helicity switches are more frequent in thinner regions of the sample, indicating a correlation between reduced volume, increased magnetic flexibility (propensity of spin textures to respond to perturbations), and helicity dynamics. The maximum switching rate reaches 2.6 switches per second in the thinner region. To provide a quantitative basis for the proposed helicity‐switching mechanism, we assume that the reversal is thermally activated. An Arrhenius analysis of the observed reversal rate of approximately $1\;s^{-1}$ in a 47nm‐thick lamella at 210K, using attempt frequencies of $10^{9}-10^{12}\;\mathrm{Hz}$ and approximately 800 candidate nucleation sites along the domain wall, gives an effective activation barrier of 0.50-0.62eV. We propose that reversal proceeds through the nucleation of a pair of magnetic defects, either vertical Bloch lines or a Bloch point–antiBloch point pair, which transiently convert a Type‐I bubble into a Type‐II configuration before the bubble reforms with opposite helicity. Using an estimated Bloch line energy of

$$\sigma_{\mathrm{BL}}=\frac{8A}{\sqrt{Q}},$$

where

$$Q=\frac{2K_{u}}{\mu_{0}M_{s}^{2}},$$

$A\approx 4.50\times 10^{-12}\;J\;m^{-1}$, $M_{s}\approx 2.8\times 10^{5}\;A\;m^{-1}$, and $K_{u}=8.31\times 10^{4}\;J\;m^{-3}$, a pair of Bloch lines spanning a 47nm‐thick lamella is estimated to cost 16.3eV, substantially exceeding the experimentally inferred activation barrier. In contrast, a simple exchange‐scale estimate of the Bloch‐point energy is

$$E_{\mathrm{BP}}\approx 12Ar^{\prime }\approx 0.11\;\mathrm{eV},$$

where $r^{\prime }=3.371\times 10^{-10}\;m$ is the average nearest‐neighbor Mn–Mn distance as calculated in the Table S3. A Bloch point–antiBloch point pair therefore costs approximately 0.23eV, which is of the same order of magnitude as the inferred activation barrier. The remaining difference may arise from domain‐wall deformation and defect nucleation and propagation. This estimate supports a thermally assisted Bloch point–antiBloch point pathway as an energetically plausible mechanism for helicity reversal.

These observations are similar to the magnetic bubble helicity switching observed by Yu et al. [68] in a [001] thin plate of hexaferrite crystal, ${\mathrm{BaFe}}_{12-x-0.005}{\mathrm{Sc}}_{x}{\mathrm{Mg}}_{0.005}O_{19}$ (BFSMO). In their study, helicity reversals in skyrmion bubbles were thermally activated by globally heating the sample near its Curie temperature, $T_{c}\approx 450$ K. At room temperature, the skyrmion bubbles coexist with the helical phase and persist to high temperatures, allowing for the study of helicity dynamics under thermal perturbation. Yu et al. reported that the helicity‐switching speed exceeded the frame rate (30 fps) of their LTEM movie, corresponding to at least 30 switches per second–significantly faster than our observation. This highlights the significant influence of thermal perturbations on helicity switching, suggesting the helicity dynamics we observe may result from weaker, more localized heating effects caused by the electron beam during LTEM imaging.

## 5 Conclusions

By tuning the magnetic anisotropy through Ga substitution in ${\mathrm{TmMn}}_{6}{\mathrm{Sn}}_{6}$, we induce a spin‐reorientation transition in ${\mathrm{TmMn}}_{6}{\mathrm{Sn}}_{4.2}{\mathrm{Ga}}_{1.8}$ and stabilize both topological (Type‐I) and non‐topological (Type‐II) magnetic bubbles. The helicity reversal in skyrmion bubbles illuminates the role of thickness in modulating the necessary interactions. This capability of leveraging chemical doping to fine‐tune anisotropy and generate a spin reorientation, transforms our ability to design and engineer topological spin textures. As we continue to investigate similar systems, different dopants or concentrations may further refine the dimensions and dynamics of magnetic bubbles, or even reveal more exotic phenomena within the broader Kagome family and beyond.

## 6 Methods

### 6.1 Crystal Growth and Structural Characterization

We grew single crystals of ${\mathrm{TmMn}}_{6}{\mathrm{Sn}}_{6-x}{\mathrm{Ga}}_{x}$ through the self‐flux method using Sn as a flux. Tm pieces (Alfa Aesar; 99.9%), Mn pieces (Alfa Aesar; 99.95%), Sn shots (Alfa Aesar; 99.999%), and Ga pieces (Alfa Aesar; X%) were added into a 2 mL aluminum oxide crucible in a molar ratio of 1: 6: 20 for ${\mathrm{TmMn}}_{6}{\mathrm{Sn}}_{6}$ and 1: 6: 15.5: 4.5 for ${\mathrm{TmMn}}_{6}{\mathrm{Sn}}_{4.2}{\mathrm{Ga}}_{1.8}$, and were sealed in a fused silica ampule under vacuum. The sealed ampule was heated to 1150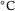 over 10 h, kept at 1150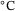 for 10 h, and then cooled to 600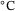 at a rate of 5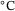/h before the tube was centrifuged. Large, hexagonal, plate‐like crystals (up to 50 mg) were obtained.

Although the initial nominal Ga concentration was x=4.5, structural and compositional analyses revealed the actual Ga content to be x=1.8, as determined through powder X‐ray diffraction (XRD) and energy‐dispersive X‐ray spectroscopy (EDX). Crystals were cleaned of residual Sn flux prior to characterization. Phase purity and structural parameters were confirmed by Rietveld refinement of powder XRD patterns collected from crushed single crystals using a Rigaku Miniflex diffractometer, with refinement carried out using the FULLPROF suite. Single‐crystal X‐ray diffraction further validated the refined lattice parameters. Elemental composition and homogeneity were confirmed via EDX mapping using a JEOL JSM‐IT500HRLV scanning electron microscope equipped with an Octane Elect Plus EDX detector. Measurements were performed at an accelerating voltage of 15 kV, with elemental ratios determined within 7% error.

### 6.2 Magnetic Measurements

We measured temperature‐dependent susceptibility with 0.1 T applied magnetic field and DC magnetization using the quantum design dynacool physical property measurement system (PPMS) with a 9‐T magnet. Both measurements were done along the ab‐plane and along the c‐axis. The AC measurement system (ACMS) option was used for both magnetization measurements.

### 6.3 Lamella Preparation

After confirming the desired composition, we prepared electron‐transparent lamellae for LTEM analysis using a ${\mathrm{Ga}}^{+}$ focused‐ion‐beam (FIB) system (Thermo Fisher Nova 200 DualBeam). After thinning the entire lamella to a thickness of 91 nm, we continued thinning half of the lamella to ≈43 nm to study the effect of thickness on spin texture stabilization. The resulting thickness profile is shown in Figure S4.

### 6.4 Lorentz Transmission Electron Microscopy

We performed the electron microscopy experiments in low‐magnification transmission electron microscopy (TEM) mode with the objective and mini‐condenser lenses turned off (Lorentz mode) in both a Talos F200X S/TEM and a JEOL NeoARM under 200 keV acceleration voltagee. For the magnetic state diagram mapping shown in Figure 3g, h, we cooled the samples to T=150K from room temperature using a Gatan 636 double tilt liquid nitrogen cooling holder and a custom‐built Gatan liquid nitrogen TEM holder with temperature control. Prior to applying a magnetic field $B_{\text{ext}}$ by exciting the objective lens current, we oriented the sample's c‐axis to be parallel with the optical axis using selected area electron diffraction. We increased $B_{\text{ext}}$ until the magnetic contrast in the LTEM image disappeared, which we interpreted as the polarization of spins into a ferromagnetic state. We then increased the temperature to the next highest one indicated by the markers in Figure 3g, h and decreased $B_{\text{ext}}$ to zero before performing a field sweep to saturation at the target temperature. We repeated this process at every temperature indicated by markers in Figure 3g, h. We used scanning transmission electron microscopy differential phase contrast (STEM‐DPC) imaging in both the Talos F200X S/TEM and JEOL NeoARM equipped with an annular pixelated detector.

The exposure time for the micrographs in Figures 2 and 3 is 5.0 s, while the exposure time for Figure 4 is 0.08 s. The mean intensity and standard deviation for a representative thin sample region in Figure 4 are $\mu_{0.08}\approx 132\;\mathrm{counts}$ and $\sigma_{0.08}=31\;\mathrm{counts}$. For the same thin plate with a 5 second exposure (from the dataset used for Figures 3, 4), $\mu_{5.0}\approx 5297\;\mathrm{counts}$ and $\sigma_{5.0}=423\;\mathrm{counts}$. Thus, the signal‐to‐noise ratio increases with exposure time: $SNR_{0.08}=4.2$ compared to $SNR_{5.0}=12.5$.

The fastest switching rates observed are approximately 5 events per frame, requiring an exposure of order 0.1 s to capture the dynamics. We also cannot exclude the possibility that we miss events because we are already close to the Nyquist frequency. Therefore, higher exposure times would not reliably capture the switching dynamics.

For the helicity switching measurements displayed in Figure 4, the integrated intensity plotted in Figure 4b is the integrated intensity of all pixels within the core of each magnetic bubble indicated in Figure 4a and defined as the circular area of pixels expanding out from the center of each bubble by a radius of 10 pixels. For visualization purposes, the integrated intensity for each bubble was normalized, Gaussian‐filtered (σ=2), and plotted with each line offset from the other. The measured mean pixel intensity and standard deviation for each bubble are listed in supplementary Table S4.

### 6.5 Thickness Mapping via Electron Energy Loss Spectroscopy

We performed electron energy loss spectroscopy on a JEOL NeoARM at 200 keV beam energy with a collection angle of β=48.2mrad. We estimated the electron's mean free path length based on the mean atomic number Z=37.29 to be λ=71.64nm, which we used to generate the thickness maps in Figure S4.

## Author Contributions

F.S.Y., N.J.G. and X.Z.Y. conceived the idea. F.S.Y. and N.J.G. coordinated the project. M.E.G., and H.B. grew single crystals. M.E.G., and R.B.R. performed structural characterizations, and physical properties measurements. F.S.Y. carried out the cryogenic LTEM and LDPC‐STEM measurements. M.E.G and J.S. independently reproduced LTEM measurements. F.S.Y., M.E.G., and N.J.G. wrote the manuscript. All authors contributed to the discussion of the results.

## Conflicts of Interest

The authors declare no conflicts of interest.

## Data and Materials Availability

The authors declare that the data supporting the findings of this study are available within the paper and its Supplementary Information files. Should any raw data files be needed in another format they are available from the corresponding author upon reasonable request.

## Supporting information


**Supporting File**: advs78269‐sup‐0001‐SuppMat.pdf.

## References

[1] A. Fert , N. Reyren , and V. Cros , “Magnetic Skyrmions: Advances in Physics and Potential Applications,” Nature Reviews Materials 2, no. 7 (2017): 1–15.

[2] A. Hirohata , K. Yamada , Y. Nakatani , et al., “Review on Spintronics: Principles and Device Applications,” Journal of Magnetism and Magnetic Materials 509 (2020): 166711.

[3] A. P. Petrovi'c , M. Raju , X. Tee , et al., “Skyrmion‐(Anti) Vortex Coupling in a Chiral Magnet‐Superconductor Heterostructure,” Physical Review Letters 126, no. 11 (2021): 117205. 10.1103/PhysRevLett.126.117205 33798341

[4] S. Mühlbauer , B. Binz , F. Jonietz , et al., “Skyrmion Lattice in a Chiral Magnet,” Science 323, no. 5916 (2009): 915–919. 10.1126/science.1166767 19213914

[5] X. Yu , Y. Onose , N. Kanazawa , et al., “Real‐Space Observation of a Two‐Dimensional Skyrmion Crystal,” Nature 465, no. 7300 (2010): 901–904. 10.1038/nature09124 20559382

[6] N. Nagaosa and Y. Tokura , “Topological Properties and Dynamics of Magnetic Skyrmions,” Nature Nanotechnology 8, no. 12 (2013): 899–911. 10.1038/nnano.2013.243 24302027

[7] G. Finocchio , J. A. C. Incorvia , J. S. Friedman , et al., “Roadmap for Unconventional Computing with Nanotechnology,” Nano Futures 8, no. 1 (2024): 012001.

[8] S. Hayami , “Skyrmion Crystals in Centrosymmetric Triangular Magnets under Hexagonal and Trigonal Single‐Ion Anisotropy,” Journal of Magnetism and Magnetic Materials 553 (2022): 169220.

[9] Y. Zhou , S. Li , X. Liang , and Y. Zhou , “Topological Spin Textures: Basic Physics and Devices,” Advanced Materials 37, no. 2 (2025): 2312935. 10.1002/adma.202312935 38861696

[10] F. Jonietz , S. Mühlbauer , C. Pfleiderer , et al., “Spin Transfer Torques in MnSi at Ultralow Current Densities,” Science 330, no. 6011 (2010): 1648–1651. 10.1126/science.1195709 21164010

[11] X. Yu , N. Kanazawa , W. Zhang , et al., “Skyrmion Flow Near Room Temperature in an Ultralow Current Density,” Nature Communications 3, no. 1 (2012): 988. 10.1038/ncomms1990 22871807

[12] T. Schulz , R. Ritz , A. Bauer , et al., “Emergent Electrodynamics of Skyrmions in a Chiral Magnet,” Nature Physics 8, no. 4 (2012): 301–304.

[13] S.‐Z. Lin , C. Reichhardt , C. D. Batista , and A. Saxena , “Particle Model for Skyrmions in Metallic Chiral Magnets: Dynamics, Pinning, and Creep,” Physical Review B 87, no. 21 (2013): 214419.

[14] Y. Zhou , “Magnetic Skyrmions: Intriguing Physics and New Spintronic Device Concepts,” National Science Review 6, no. 2 (2019): 210–212. 10.1093/nsr/nwy109 PMC8294180 34691858

[15] M. Ezawa , “Giant Skyrmions Stabilized by Dipole‐Dipole Interactions in Thin Ferromagnetic Films,” Physical Review Letters 105, no. 19 (2010): 197202. 10.1103/PhysRevLett.105.197202 21231193

[16] J. T. Lee , J. Chess , S. Montoya , et al., “Synthesizing Skyrmion Bound Pairs in Fe‐Gd Thin Films,” Applied Physics Letters 109, no. 2 (2016).

[17] S. Woo , K. M. Song , X. Zhang , et al., “Current‐Driven Dynamics and Inhibition of the Skyrmion Hall Effect of Ferrimagnetic Skyrmions in GdFeCo Films,” Nature Communications 9, no. 1 (2018): 959. 10.1038/s41467-018-03378-7 PMC5840382 29511179

[18] J. Sampaio , V. Cros , S. Rohart , A. Thiaville , and A. Fert , “Nucleation, Stability and Current‐Induced Motion of Isolated Magnetic Skyrmions in Nanostructures,” Nature Nanotechnology 8, no. 11 (2013): 839–844. 10.1038/nnano.2013.210 24162000

[19] W. Jiang , P. Upadhyaya , W. Zhang , et al., “Blowing Magnetic Skyrmion Bubbles,” Science 349, no. 6245 (2015): 283–286. 10.1126/science.aaa1442 26067256

[20] A. Soumyanarayanan , M. Raju , A. Gonzalez Oyarce , et al., “Tunable Room‐Temperature Magnetic Skyrmions in Ir/Fe/Co/Pt Multilayers,” Nature Materials 16, no. 9 (2017): 898–904. 10.1038/nmat4934 28714983

[21] S. Hayami , S.‐Z. Lin , and C. D. Batista , “Bubble and Skyrmion Crystals in Frustrated Magnets with Easy‐Axis Anisotropy,” Physical Review B 93, no. 18 (2016): 184413.

[22] S. Hayami and Y. Motome , “Square Skyrmion Crystal in Centrosymmetric Itinerant Magnets,” Physical Review B 103, no. 2 (2021): 024439.

[23] J. A. Paddison , B. K. Rai , A. F. May , et al., “Magnetic Interactions of the Centrosymmetric Skyrmion Material ${\mathrm{Gd}}_{2}{\mathrm{PdSi}}_{3}$ ,” Physical Review Letters 129, no. 13 (2022): 137202. 10.1103/PhysRevLett.129.137202 36206423

[24] S. Zuo , K. Qiao , Y. Zhang , T. Zhao , C. Jiang , and B. Shen , “Spontaneous Biskyrmion Lattice in a Centrosymmetric Rhombohedral Rare‐Earth Magnet with Easy‐Plane Anisotropy,” Nano Letters 23, no. 2 (2023): 550–557. 10.1021/acs.nanolett.2c04001 36633430

[25] D. Chakrabartty , S. Jamaluddin , S. K. Manna , and A. K. Nayak , “Tunable Room Temperature Magnetic Skyrmions in Centrosymmetric Kagome Magnet ${\mathrm{Mn}}_{4}{\mathrm{Ga}}_{2}\mathrm{Sn}$ ,” Communications Physics 5, no. 1 (2022): 189.

[26] Y. Tokura and N. Kanazawa , “Magnetic Skyrmion Materials,” Chemical Reviews 121, no. 5 (2020): 2857–2897. 10.1021/acs.chemrev.0c00297 33164494

[27] M. Hirschberger , S. Hayami , and Y. Tokura , “Nanometric Skyrmion Lattice from Anisotropic Exchange Interactions in a Centrosymmetric Host,” New Journal of Physics 23, no. 2 (2021): 023039.

[28] T. Okubo , S. Chung , and H. Kawamura , “Multiple‐q States and the Skyrmion Lattice of the Triangular‐Lattice Heisenberg Antiferromagnet under Magnetic Fields,” Physical Review Letters 108, no. 1 (2012): 017206. 10.1103/PhysRevLett.108.017206 22304286

[29] A. O. Leonov and M. Mostovoy , “Multiply Periodic States and Isolated Skyrmions in an Anisotropic Frustrated Magnet,” Nature Communications 6 (2015): 8275. 10.1038/ncomms9275 PMC4667438 26394924

[30] V. Ahrens , C. Kiesselbach , L. Gnoli , et al., “Skyrmions Under Control–FIB Irradiation as a Versatile Tool for Skyrmion Circuits,” Advanced Materials 35, no. 2 (2023): 2207321, 10.1002/adma.202207321. 36255142

[31] L.‐M. Kern , V. M. Kuchkin , V. Deinhart , et al., “Controlled Formation of Skyrmion Bags,” Advanced Materials 37, no. 29 (2025): 2501250, 10.1002/adma.202501250. PMC12288806 40255099

[32] W. Kang , Y. Huang , X. Zhang , Y. Zhou , and W. Zhao , “Skyrmion‐Electronics: An Overview and Outlook,” Proceedings of the IEEE 104, no. 10 (2016): 2040–2061.

[33] I. Martin and A. F. Morpurgo , “Majorana Fermions in Superconducting Helical Magnets,” Physical Review B 85, no. 14 (2012): 144505.

[34] F. Freimuth , R. Bamler , Y. Mokrousov , and A. Rosch , “Phase‐Space Berry Phases in Chiral Magnets: Dzyaloshinskii‐Moriya Interaction and the Charge of Skyrmions,” Physical Review B 88, no. 21 (2013): 214409.

[35] J. L. Lado and J. Fernández‐Rossier , “Quantum Anomalous Hall Effect in Graphene Coupled to Skyrmions,” Physical Review B 92, no. 11 (2015): 115433.

[36] N. Paul and L. Fu , “Topological Magnetic Textures in Magnetic Topological Insulators,” Physical Review Research 3, no. 3 (2021): 033173.

[37] A. P. Petrovi'c , C. Psaroudaki , P. Fischer , M. Garst , and C. Panagopoulos , “Colloquium: Quantum Properties and Functionalities of Magnetic Skyrmions,” arXiv (2024): arXiv:2410.11427.

[38] G. E. Volovik , The Universe in a Helium Droplet, vol. 117 (OUP Oxford, 2003).

[39] G. Volovik , “Interplay of Real Space and Momentum Space Topologies in Strongly Correlated Fermionic Systems,” in Topology of Strongly Correlated Systems (World Scientific, 2001), 30–49.

[40] N. Nagaosa , X. Yu , and Y. Tokura , “Gauge Fields in Real and Momentum Spaces in Magnets: Monopoles and Skyrmions,” Philosophical Transactions of the Royal Society A 370, no. 1981 (2012): 5806–5819. 10.1098/rsta.2011.0405 23166382

[41] Z. Wang , Y. Su , S.‐Z. Lin , and C. D. Batista , “Skyrmion Crystal from RKKY Interaction Mediated by 2D Electron Gas,” Physical Review Letters 124, no. 20 (2020): 207201. 10.1103/PhysRevLett.124.207201 32501075

[42] K. Karube , L. Peng , J. Masell , et al., “Doping Control of Magnetic Anisotropy for Stable Antiskyrmion Formation in Schreibersite (Fe,Ni) with S4 Symmetry,” Advanced Materials 34, no. 11 (2022): 2108770. 10.1002/adma.202108770 35032408

[43] T. Kurumaji , T. Nakajima , M. Hirschberger , et al., “Skyrmion Lattice with a Giant Topological Hall Effect in a Frustrated Triangular‐Lattice Magnet,” Science 365, no. 6456 (2019): 914–918. 10.1126/science.aau0968 31395744

[44] N. D. Khanh , T. Nakajima , X. Yu , et al., “Nanometric Square Skyrmion Lattice in a Centrosymmetric Tetragonal Magnet,” Nature Nanotechnology 15, no. 6 (2020): 444–449. 10.1038/s41565-020-0684-7 32424341

[45] M. Hirschberger , T. Nakajima , S. Gao , et al., “Skyrmion Phase and Competing Magnetic Orders on a Breathing Kagomé Lattice,” Nature Communications 10, no. 1 (2019): 5831. 10.1038/s41467-019-13675-4 PMC6930224 31874953

[46] M. Kakihana , D. Aoki , A. Nakamura , et al., “Unique Magnetic Phases in the Skyrmion Lattice and Fermi Surface Properties in Cubic Chiral Antiferromagnet EuPtSi,” Journal of the Physical Society of Japan 88, no. 9 (2019): 094705.

[47] J. M. Moya , S. Lei , E. M. Clements , et al., “Incommensurate Magnetic Orders and Topological Hall Effect in the Square‐Net Centrosymmetric ${\mathrm{EuGa}}_{2}{\mathrm{Al}}_{2}$ System,” Physical Review Materials 6, no. 7 (2022): 074201.

[48] H. Zhang , X. Zhu , Y. Xu , et al., “Giant Magnetoresistance and Topological Hall Effect in the ${\mathrm{EuGa}}_{4}$ Antiferromagnet,” Journal of Physics: Condensed Matter 34, no. 3 (2021): 034005. 10.1088/1361-648X/ac3102 34666329

[49] M. Gen , R. Takagi , Y. Watanabe , et al., “Rhombic Skyrmion Lattice Coupled with Orthorhombic Structural Distortion in ${\mathrm{EuAl}}_{4}$ ,” Physical Review B 107, no. 2 (2023): L020410.

[50] N. J. Ghimire , R. L. Dally , L. Poudel , et al., “Competing Magnetic Phases and Fluctuation‐Driven Scalar Spin Chirality in the Kagome Metal ${\mathrm{YMn}}_{6}{\mathrm{Sn}}_{6}$ ,” Science Advances 6, no. 51 (2020): eabe2680. 10.1126/sciadv.abe2680 PMC11206209 33355145

[51] D. C. Jones , S. Das , H. Bhandari , et al., “Origin of Spin Reorientation and Intrinsic Anomalous Hall Effect in the Kagome Ferrimagnet ${\mathrm{TbMn}}_{6}{\mathrm{Sn}}_{6}$ ,” Physical Review B 110, no. 11 (2024): 115134.

[52] P. E. Siegfried , H. Bhandari , D. C. Jones , et al., “Magnetization‐Driven Lifshitz Transition and Charge‐Spin Coupling in the Kagome Metal ${\mathrm{YMn}}_{6}{\mathrm{Sn}}_{6}$ ,” Communications Physics 5, no. 1 (2022): 58.

[53] Z. Li , Q. Yin , Y. Jiang , et al., “Discovery of Topological Magnetic Textures Near Room Temperature in Quantum Magnet ${\mathrm{TbMn}}_{6}{\mathrm{Sn}}_{6}$ ,” Advanced Materials 35, no. 20 (2023): 2211164. 10.1002/adma.202211164 36856016

[54] G. Venturini , D. Fruchart , and B. Malaman , “Incommensurate Magnetic Structures of ${\mathrm{RMn}}_{6}{\mathrm{Sn}}_{6}$ (R = Sc, Y, Lu) Compounds from Neutron Diffraction Study,” Journal of Alloys and Compounds 236, no. 1‐2 (1996): 102–110.

[55] F. Canepa , M. Napoletano , C. Lefèvre , and G. Venturini , “A Magnetisation Study of ${\mathrm{TmMn}}_{6}{\mathrm{Sn}}_{6-x}{\mathrm{Ga}}_{x}$ Single Crystals (0.15≤x≤1.90),” Journal of Magnetism and Magnetic Materials 285, no. 1‐2 (2005): 254–266.

[56] M. E. Gazzah , P.‐H. Chang , Y. Lee , et al., “Doping‐Induced Spin Reorientation in Kagome Magnet ${\mathrm{TmMn}}_{6}{\mathrm{Sn}}_{6}$ ,” Physical Review Materials 9 (2025): 114414.

[57] X. Yu , M. Mostovoy , Y. Tokunaga , et al., “Magnetic Stripes and Skyrmions with Helicity Reversals,” Proceedings of the National Academy of Sciences 109, no. 23 (2012): 8856–8860. 10.1073/pnas.1118496109 PMC3384203 22615354

[58] C. Kittel , “Theory of the Structure of Ferromagnetic Domains in Films and Small Particles,” Physical Review 70, no. 11‐12 (1946): 965.

[59] M. Heigl , S. Koraltan , M. Vaňatka , et al., “Dipolar‐Stabilized First and Second‐Order Antiskyrmions in Ferrimagnetic multilayers,” Nature Communications 12, no. 1 (2021): 2611. 10.1038/s41467-021-22600-7 PMC8110839 33972515

[60] M. Hassan , S. Koraltan , A. Ullrich , et al., “Dipolar Skyrmions and Antiskyrmions of Arbitrary Topological Charge at Room Temperature,” Nature Physics 20, no. 4 (2024): 615–622. 10.1038/s41567-023-02358-z PMC11021192 38638455

[61] E. M. Jefremovas , K. Leutner , M. G. Fischer , et al., “The Role of Magnetic Dipolar Interactions in Skyrmion Lattices,” arXiv (2024): arXiv:2407.00539. 10.1016/j.newton.2025.100036 PMC11976062 40206846

[62] I. Žutić , J. Fabian , and S. D. Sarma , “Spintronics: Fundamentals and Applications,” Reviews of Modern Physics 76, no. 2 (2004): 323.

[63] S. Luo and L. You , “Skyrmion Devices for Memory and Logic Applications,” APL Materials 9, no. 5 (2021).

[64] L. Kong , J. Tang , Y. Wu , et al., “Diverse Helicities of Dipolar Skyrmions,” Physical Review B 109, no. 1 (2024): 014401.

[65] M.‐G. Han , J. D. Thomsen , J. P. Philbin , et al., “Electric Field Control of Magnetic Skyrmion Helicity in a Centrosymmetric 2D van der Waals Magnet,” Nano Letters 25, no. 13 (2025): 5174–5180, 10.1021/acs.nanolett.4c06200, PMID: 40067769 . 40067769

[66] R. Egerton and S. Cheng , “Measurement of Local Thickness by Electron Energy‐Loss Spectroscopy,” Ultramicroscopy 21, no. 3 (1987): 231–244.

[67] F. S. Yasin , J. Masell , Y. Takahashi , et al., “Bloch Point Quadrupole Constituting Hybrid Topological Strings Revealed with Electron Holographic Vector Field Tomography,” Advanced Materials 36, no. 16 (2024): 2311737, 10.1002/adma.202311737. 38219021

[68] X. Yu , K. Shibata , W. Koshibae , et al., “Thermally Activated Helicity Reversals of Skyrmions,” Physical Review B 93, no. 13 (2016): 134417.

