# Supplementary material for: Skyrmion Bubbles by Design in a Centrosymmetric Kagome Magnet
Source: Adv Sci (Weinh). 2026 Oct 20:e78269. Online ahead of print. doi: 10.1002/advs.78269 (PMC13683820; doi:10.1002/advs.78269)
Supplement: Supplementary file 1 [file ADVS-9999-e78269-s001.pdf]

Supplemental information for:
Skyrmion Bubbles by Design in a Centrosymmetric Kagome Magnet

Mohamed El Gazzah,[1,2,*] **Fehmi Sami Yasin**,[3,4,†] **Sk Jamaluddin**,[1,2] **Hari Bhandari**,[1,2,5] **Resham Babu Regmi**,[1,2] **Xiuzhen Yu**,[4,6] and **Nirmal J. Ghimire**[‡]

[1]*Department of Physics and Astronomy, University of Notre Dame, Notre Dame, IN 46556, USA*

[2]*Stavropoulos Center For Complex Quantum Matter, University of Notre Dame, Notre Dame, IN 46556, USA*

[3]*Center for Nanophase Materials Sciences, Oak Ridge National Laboratory, Oak Ridge, TN 37830, USA*

[4]*RIKEN Center for Emergent Matter Science, Wako 351-0198, Japan*

[5]*Department of Physics and Astronomy, Rice University, Houston, Texas 77005, USA*

[6]*The Institute of Science Tokyo, Tokyo 152-8550, Japan*

(Dated: August 31, 2026)

S1. CRYSTAL STRUCTURE AND POWDER X-RAY DIFFRACTION ANALYSIS

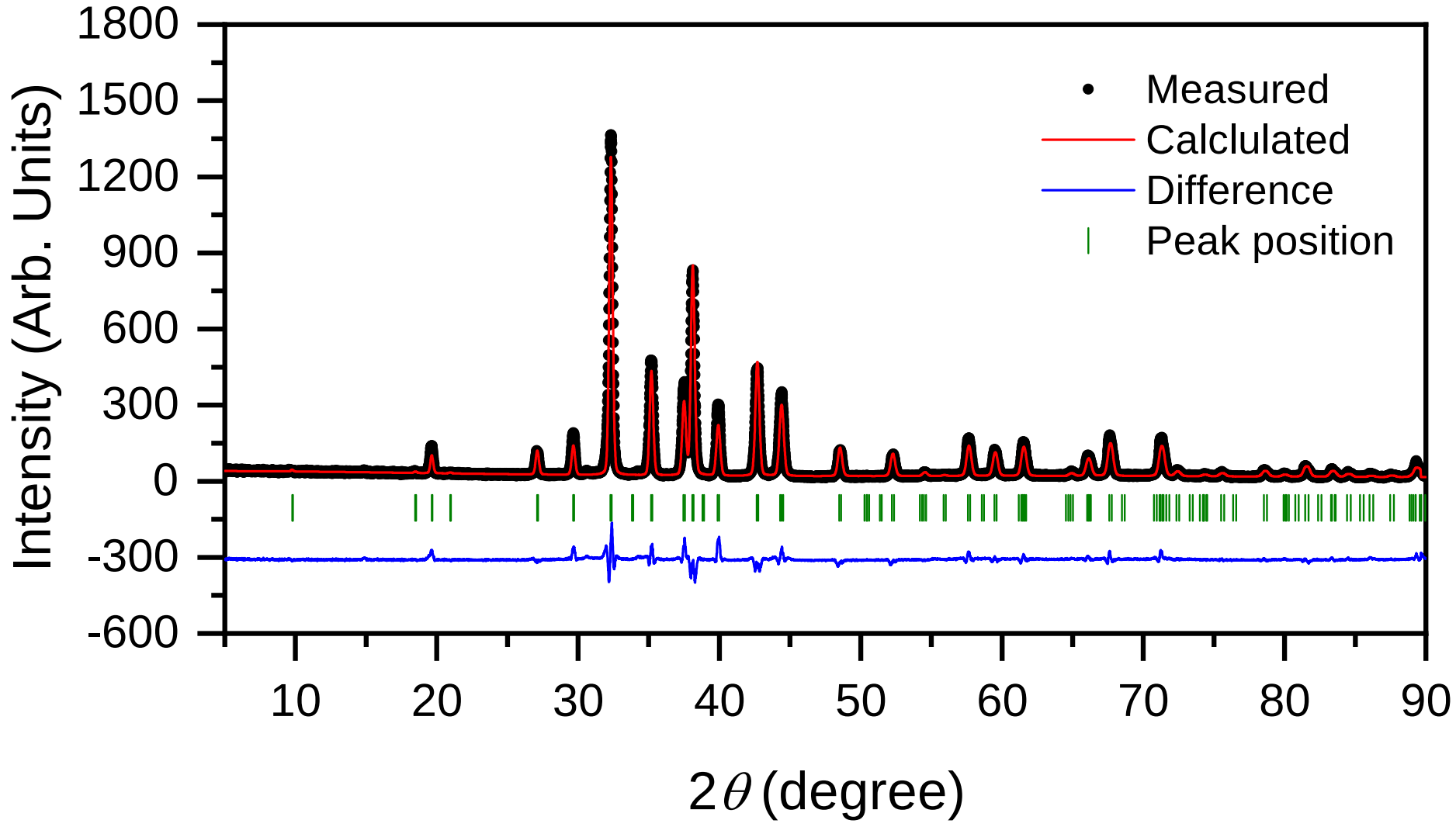

FIG. S1 : Structural Characterization. Rietveld refinement of the X-ray powder pattern of $TmMn_6Sn_{4.2}Ga_{1.8}$ measured at room temperature.

TABLE S1 : Selected data from Rietveld refinement of powder X-ray diffraction collected on ground crystals of $TmMn_6Sn_{4.2}Ga_{1.8}$.

Space group	*P6/mmm*
Unit cell a, c (Å)	5.40848(8), 8.82625(17)
R_{WP}	11.7 %
R_B	5.39 %
R_F	5.28 %

Atom	Position	x	y	z
Tm	1a	0	0	0
Mn	6i	0	1/2	0.24080(19)
Sn_1	2e	0	0	0.33495(18)
Sn_2	2d	1/3	2/3	1/2
Sn_3	2c	1/3	2/3	0
Ga	2c	1/3	2/3	0

The crystal structure of $TmMn_6Sn_{4.2}Ga_{1.8}$ was confirmed through Rietveld refinement of powder X-ray diffraction (PXRD) data collected at room temperature using a Rigaku MiniFlex diffractometer. A representative single crystal was cleaned to remove residual surface flux and then ground into a fine powder for diffraction measurements. Rietveld refinements were performed using the FULLPROF software package[1]. The refinement is shown in Fig. S1, with detailed crystallographic parameters summarized in Table S1.

The analysis revealed that Ga substitution occurs specifically at the Sn3 site (Wyckoff position 2c), as modeling Ga occupancy at alternative Sn positions led to unphysical occupancies or poor refinement fits. This targeted substitution was further supported by chemical composition analysis using energy-dispersive X-ray spectroscopy (EDS), which confirmed a stoichiometry close to $TmMn_6Sn_{4.2}Ga_{1.8}$ with less than 7% error.

S2. MAGNETIZATION AND ANISOTROPY CALCULATION

Magnetic susceptibility and magnetization measurements were carried out using a Quantum Design Dynacool Physical Property Measurement System (PPMS) with a 9-T magnet. The AC Measurement System (ACMS) option was used for these measurements. Orientation of each crystal was checked by measuring the (001) peak from the surface of the crystal using the Rigaku Miniflex X-ray diffractometer.

The anisotropy constant K_u was estimated by integrating the area enclosed between the magnetization curves measured along the c-axis and in-plane directions, as shown in Figure S2. At temperatures where the saturation field exceeded our accessible field range, we extrapolated the hard-axis magnetization curve until it intersected the saturation magnetization of the easy axis. This extrapolation is shown in Figs. S2g-h.

Magnetization was converted from $(\mu_{\rm B})$/F.U to A/m using $(M = n\mu_{\rm B}/V_{\rm F.U})$ with $V_{\rm fu}$ calculated from the lattice parameters. The difference $(\Delta M(H) = M_{\rm easy}(H) - M_{\rm hard}(H))$, highlighted in green, was integrated. With that convention, $K_u = \int \Delta M, {\rm d}(\mu_0 H),$.

While this extrapolation introduces some uncertainty, it provides a reasonable quantitative estimate of how the anisotropy evolves with temperature, complementing the qualitative trends already inferred from the susceptibility and magnetization measurements.

The standard deviation in the magnetization measurements is 0.1% as averaged over the entire range. In calculating the error of the anisotropy shown in Fig. 1f, we utilized the

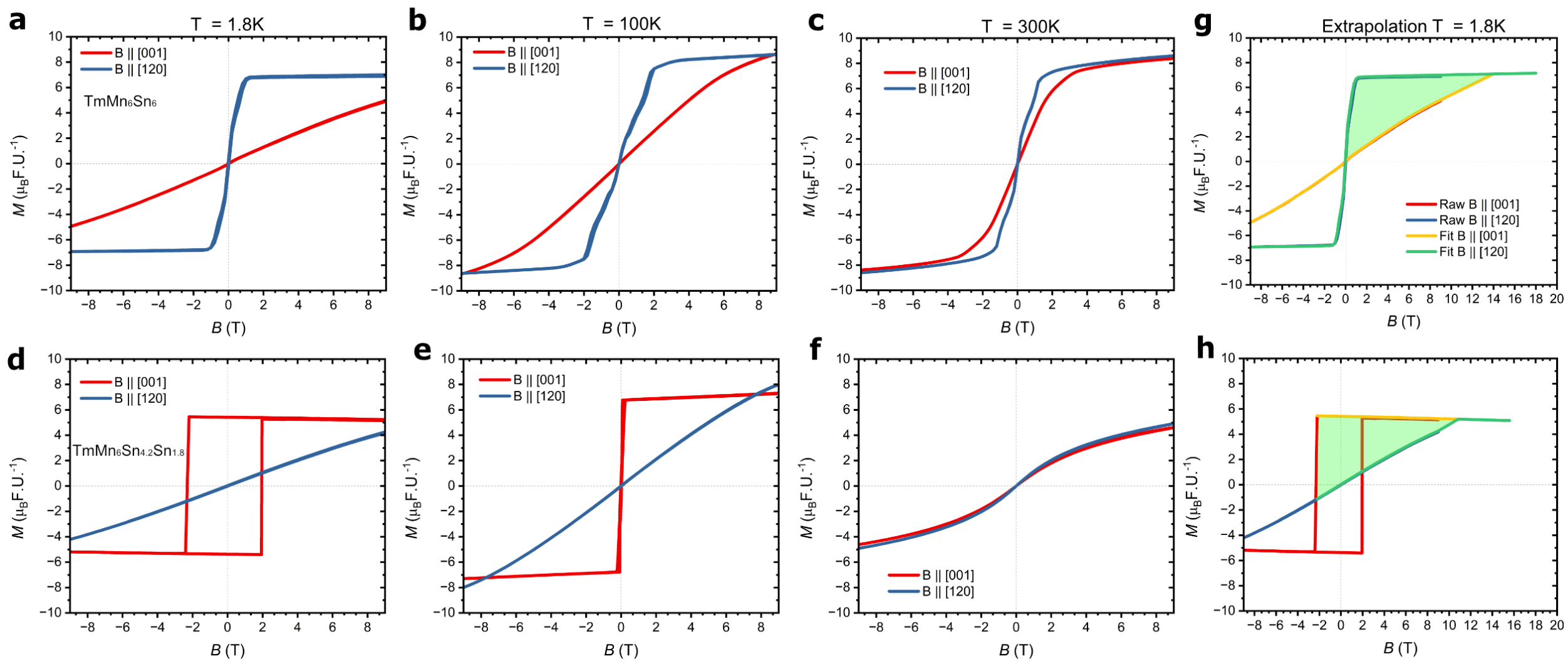

FIG. S2 : Magnetization curves and magnetocrystalline anisotropy energy determination for $TmMn_6Sn_6$ and $TmMn_6Sn_{4.2}Sn_{1.8}$. M–H curves measured along B || [001] (red) and B || [120] (blue) directions for (a–c) $TmMn_6Sn_6$ and (d–f) $TmMn_6Sn_{4.2}Sn_{1.8}$ at T = 1.8 K, 100 K, and 300 K, respectively. For $TmMn_6Sn_6$, the in-plane magnetization saturates more rapidly than the out-of-plane direction, evidencing the easy-plane anisotropy, while for $TmMn_6Sn_{4.2}Sn_{1.8}$, the c-axis magnetization saturates more rapidly confirming the c-axis anisotropy at, and below 200 K. (g, h) Extrapolation procedure used to estimate the anisotropy constant K_u at T = 1.8 K for $TmMn_6Sn_6$ and $TmMn_6Sn_{4.2}Sn_{1.8}$, respectively. The raw M-H curves (red and blue) are extended beyond the measurement range (orange and green fits) until the hard-axis fit reaches the saturation magnetization defined by the easy axis. The magnetocrystalline anisotropy energy K_u is obtained by integrating the area enclosed between the extrapolated easy- and hard-axis curves (shaded green region).

following standard propagation of error equation:

$$\sigma_{K_u} = \frac{2\mu_B}{V_{\text{F.U.}}}\sqrt{\sum w_k^2 \sigma_m^2} \tag{S1}$$

where σ_m is the measurement error (0.1%), and w_k represents the sensitivity weights of the trapezoidal sum to the integrand of the difference between the easy- and hard-axis magnetizations. The boundary weight values are $w_{\text{initial}} = w_{\text{final}} = 0.0064$ T, while the interior values are 0.0129 T. This yields a maximum propagated error of 1% at 200 K. Due to the negligible magnitude of this error, the error bars have been omitted from the Fig. 1f

plot.

TABLE S2 : Magnetic parameters of $TmMn_6Sn_6$ and $TmMn_6Sn_{4.2}Ga_{1.8}$ at selected temperatures.

Sample	T (K)	K_u ($\mathrm{J\,m^{-3}}$)	M_s (μ_B/f.u.)	M_s ($\mathrm{A\,m^{-1}}$)	Q
$TmMn_6Sn_6$	1.8	-1.60×10^6	6.80	2.67×10^5	−35.76
$TmMn_6Sn_6$	300	-2.45×10^5	8.23	3.23×10^5	−3.75
$TmMn_6Sn_{4.2}Ga_{1.8}$	1.8	1.77×10^6	5.45	2.26×10^5	54.83
$TmMn_6Sn_{4.2}Ga_{1.8}$	200	8.31×10^4	6.73	2.79×10^5	1.69

S3. MEAN FIELD EXCHANGE STIFFNESS CALCULATION

In its full form, the exchange stiffness energy is

$$A_\nu(0\text{ K}) = \frac{1}{V_0}\sum_{ij}\frac{J_\nu^{ij}}{2}\left(a_\nu^{ij}\right)^2, \tag{S2}$$

where $\nu = x, y, z$, V_0 is the unit-cell volume, and J_ν^{ij} is the site-resolved exchange energy between spins i and j for a given axis or plane: the in-plane (x–y) component corresponds to the intralayer Mn–Mn exchange, while the z component contains both the interlayer Mn–Mn exchange and the Tm–Mn exchange. Equation (S2) is equivalent to the real-space expression for the spin-wave stiffness given by Pajda et al[2].

To resolve the exchange stiffness we may either use the exchange energies directly, as obtained from density functional theory, or approximate them through the Curie temperature. For an isotropic lattice with nearest-neighbour interactions the classical mean-field Curie temperature gives

$$J = \frac{3k_BT_C}{z}, \tag{S3}$$

where z is the number of nearest neighbours, T_C is the Curie temperature and k_B is the Boltzmann constant.

Within the mean-field approximation we assume that the interaction across every bond is identical, so that for cubic symmetry or higher

$$A \;=\; \frac{n_s}{12}\sum_i J_i\, r_i^2, \tag{S4}$$

where n_s is the magnetic-atom density.

We now define an average displacement r', which takes the place of the individual spacing r_i of every pair of spins. The unit cell contains two kagome layers of Mn, each with three Mn atoms; Mn occupies the Wyckoff $6i$ site $(0, \frac{1}{2}, z_{\mathrm{Mn}})$ with $z_{\mathrm{Mn}} = 0.24379$. Each Mn atom has four in-plane nearest neighbours, and for the Mn sitting at $(a/2, 0)$ the four neighbour displacements are

$$\mathbf{r}_i = \left(\pm\frac{a}{4}, \pm\frac{\sqrt{3}}{4}a\right), \qquad |\mathbf{r}_i| = \frac{a}{2}. \tag{S5}$$

Each Mn additionally has one neighbour directly above and one directly below, since the kagome nets are stacked along c, at

$$d_1 = c\,(1 - 2z_{\mathrm{Mn}}) \quad \text{and} \quad d_2 = 2z_{\mathrm{Mn}}c, \tag{S6}$$

which cross the Sn ($2d$) and the Tm/Ga ($2c$) layers respectively. The total coordination is therefore $z = 6$, and the average displacement is

$$(r')^2 \;=\; \frac{1}{6}\sum_i r_i^2 \;=\; \frac{4\,(a/2)^2 + d_1^2 + d_2^2}{6}. \tag{S7}$$

For classical spins,

$$3k_BT_C \;=\; \sum_i J_i, \tag{S8}$$

so that Eq. (S4) becomes

$$A \;=\; \frac{n_s}{12}\,(r')^2\,\big(3k_BT_C\big), \tag{S9}$$

and therefore

$$\boxed{A \;=\; \frac{n_{\mathrm{Mn}}\,k_BT_C\,(r')^2}{4}} \tag{S10}$$

The unit-cell parameters change with Ga concentration as tracked in Fig. 3 of Ref.[3]. That data is reproduced in the table below, together with the Mn density $n_{\mathrm{Mn}} = 6/V_0$ (six Mn per formula unit, $Z = 1$, with $V_0 = \frac{\sqrt{3}}{2}a^2c$), the average displacement of Eq. (S7), and the resulting exchange stiffness of Eq. (S10).

x	T_C (K)	a (Å)	c (Å)	V (Å^3)	n (10^{28} m^{-3})	r' (Å)	A (pJ m^{-1})
0	347	5.515	8.978	236.5	2.537	3.434	**3.58**
0.2	370	5.504	8.960	235.0	2.553	3.427	**3.83**
1.2	335	5.447	8.869	227.9	2.633	3.392	**3.50**
1.8	280	5.414	8.814	223.7	2.682	3.371	**2.95**

TABLE S3 **:** Exchange stiffness obtained from Eq. (S10). The variation across the full doping series is below 20% and is non-monotonic, tracking T_C: the exchange stiffness is therefore only weakly affected by Ga substitution.

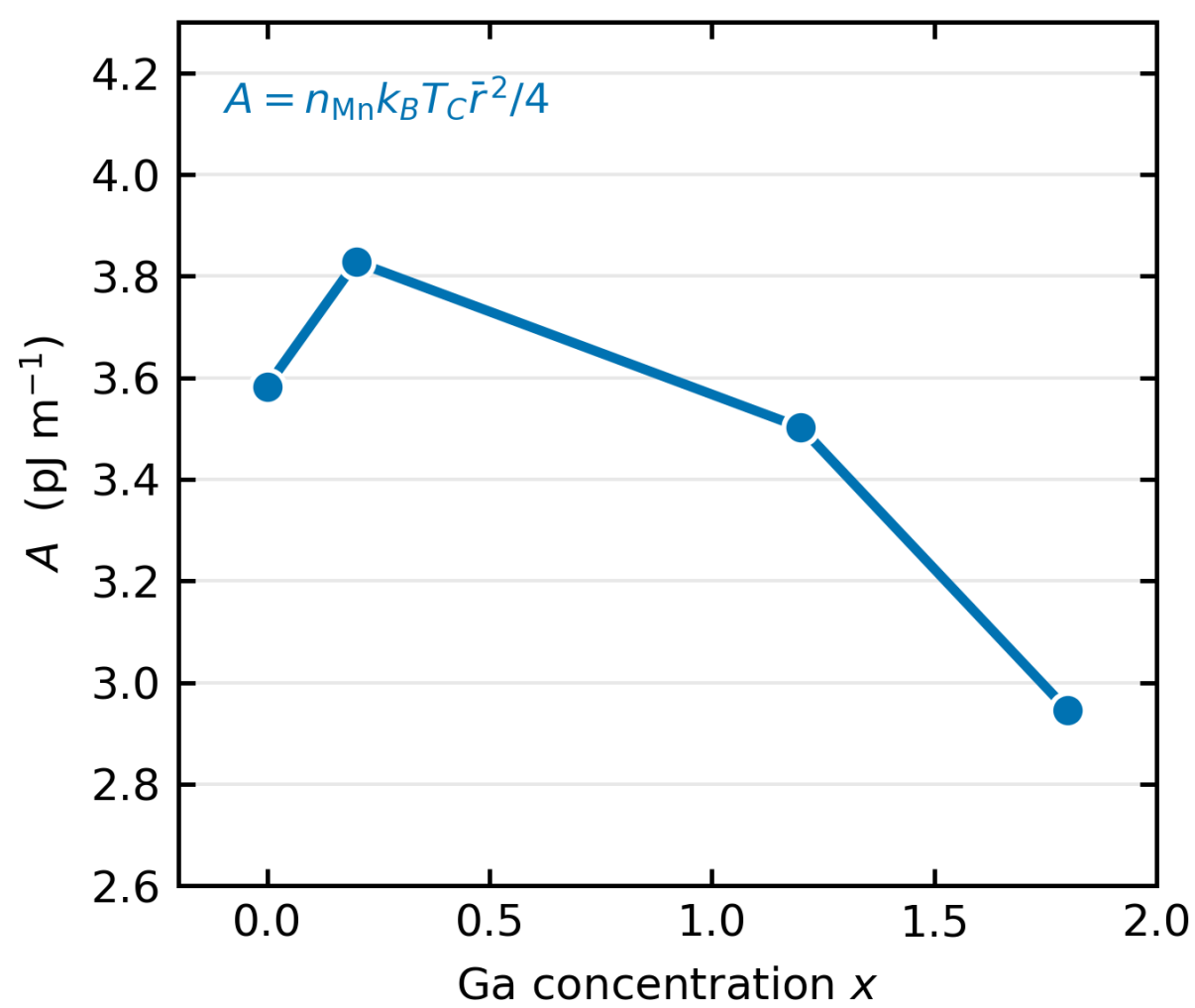

FIG. S3 : Exchange stiffness A of $TmMn_6Sn_{6-x}Ga_x$ as a function of Ga concentration x, obtained within the mean-field approximation from the measured Curie temperatures using $A = n_{\mathrm{Mn}} k_B T_C (r')^2/4$ [Eq. (S10)].

S4. HELICITY SWITCHING ANALYSIS

We observed helicity switching in Type-I skyrmionic bubbles, which was found to be thickness-dependent. Using in situ temperature control in the LTEM, the switching behavior was tracked across a range of temperatures below the spin reorientation temperature (243 K). We confirmed helicity switching by observing the gradual, reversible rotation of magnetic domain contrast at zero magnetic field. The helicity-switching rate was inversely proportional to lamella thickness, suggesting a potential sensitivity to sample dimensions, likely due to variations in local anisotropy and dipolar interactions. Further analysis of this

TABLE S4 **:** Mean Intensity within the Magnetic Bubbles Core Area (radius = 10 pixels, 1 frame).

1: 101 ± 3	2: 105 ± 4	3: 107 ± 4	4: 111 ± 3	5: 124 ± 3
6: 118 ± 4	7: 101 ± 3	8: 128 ± 3	9: 129 ± 5	10: 134 ± 3
11: 146 ± 3	12: 129 ± 9	13: 137 ± 3	14: 142 ± 4	15: 147 ± 4
16: 156 ± 4	17: 125 ± 3	18: 118 ± 2	19: 177 ± 5	20: 172 ± 4
21: 161 ± 4	22: 154 ± 6	23: 144 ± 6	24: 124 ± 3	25: 126 ± 3
26: 137 ± 4	27: 175 ± 8	28: 166 ± 8	29: 162 ± 8	

behavior is shown in Fig. 4.

S5. PHASE DIAGRAM DETAILS

To construct the phase diagrams presented in the main text, magnetic field sweeps were conducted at 265 K, 260 K, 250 K, 245 K, 240 K, 230 K, 200 K, and 150 K from 0 mT to saturation. The diagrams were created based on observations of the real space magnetic contrast under increasing magnetic field along the c-axis, using LTEM imaging. Fig. S5 shows selected real space magnetic images acquired over the temperature and magnetic field ranges noted above. A clear change in magnetic contrast in the LTEM images in Fig. S5 can be noted near the spin reorientation temperature, as described in the main text.

We measured the magnetic bubble densities by counting the number of bubbles in the thin and thick region of the lamella and dividing by the area of each region, 7.1 μm^2 and 6.8 μm^2, respectively at each magnetic field and temperature condition. As described in the main text, we then scaled the resulting density in the thicker region by 2.12: $\rho^*_{\text{bubbles}}(d = 91 \text{ nm}) = D(d = 91 \text{ nm}) \times D(d = 43 \text{ nm})/\rho_{\text{bubbles}}(d = 91 \text{ nm}) \propto 91 \text{ nm}/43 \text{ nm} = \rho_{\text{bubbles}}(d = 91 \text{ nm}) \times 2.12$ to account for the difference in bubble density attributable to the difference in thickness[4]. In that work, the total energy of a thin film magnet with uniaxial anisotropy per unit area is derived to be $F = \sigma_w \left[2\sqrt{2} + (d - D)/D\right] + \varepsilon_{\text{a}} D/2$, where σ_w is t he surface energy density of the Bloch wall, ε_{a} is the anisotropy energy density, d is the film thickness, and D is the characteristic domain size. The energy is minimum when $D = (2\sigma_w d/\varepsilon_{\text{a}})^{1/2}$. In our case, Fig. S6 provides a comparison of the domain sizes between the 43 nm and 91 nm thick lamellae. Using the ground magnetic states after zero field cooling to $T = 150$ K,

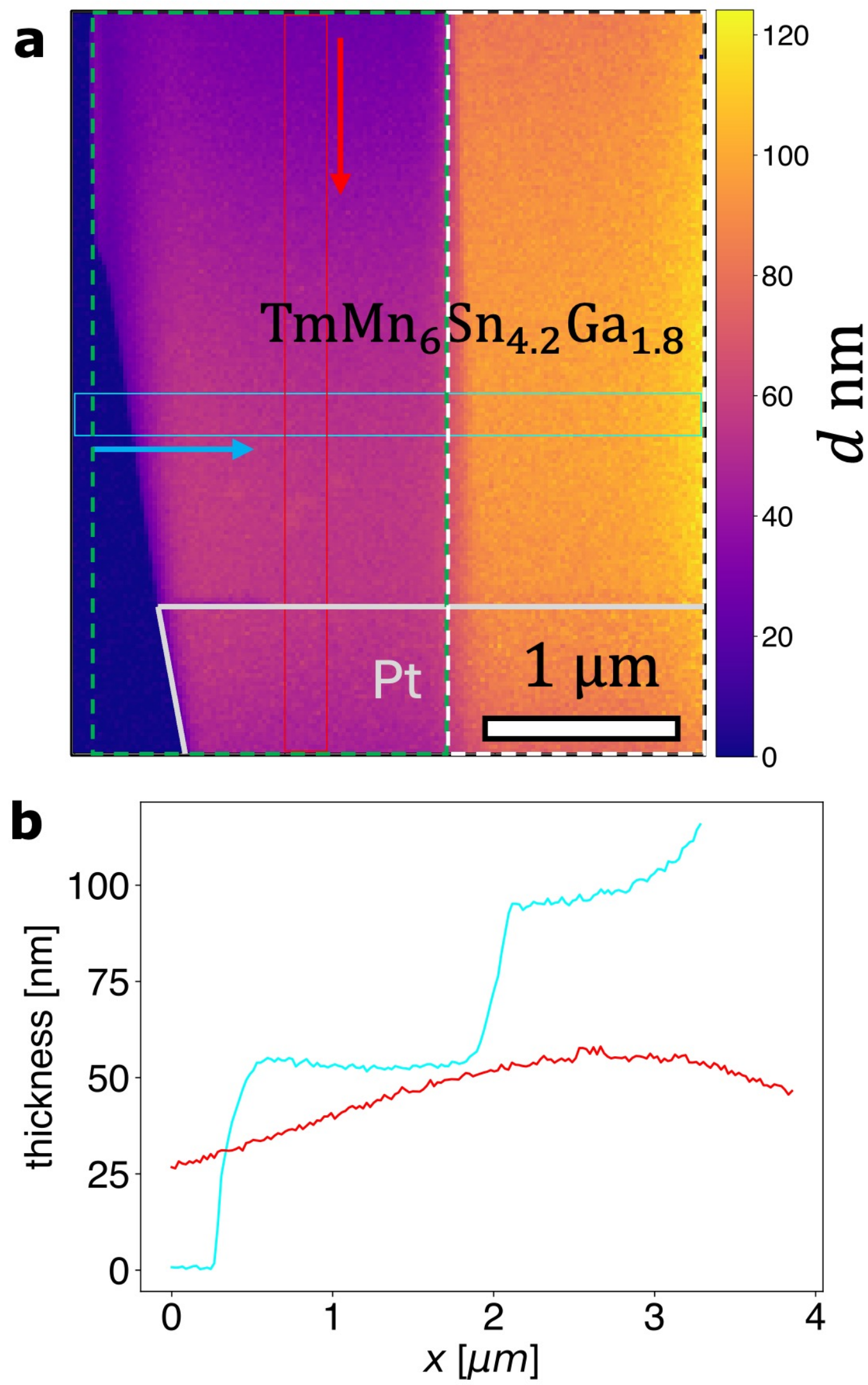

FIG. S4 : Thickness mapping of a $TmMn_6Sn_{4.2}Ga_{1.8}$ lamella using electron energy loss spectroscopy (EELS), highlighting spatial variation across the sample. (**A**) EELS image of the TEM sample studied, where (**B**) the plot shows vertical (red) and horizontal (blue) thickness profiles relative to distance from the lamella edge. The blue line displays a step-like variation due to the wedge-shaped preparation of the sample, while the red line remains relatively constant, representing the vertical thickness across the lamella. The average thicknesses in the thin and thick regions(excluding pixels with $d < 5$ nm) outlined by a green and white dashed rectangle, respectively, are $\overline{d}_{\text{thin}} = 43 \pm 10$ nm and $\overline{d}_{\text{thick}} = 91 \pm 8$ nm.

we determined that the domain sizes are $D_{43\ \text{nm}} = 85 \pm 17$ nm and $D_{91\ \text{nm}} = 124 \pm 12$ nm,

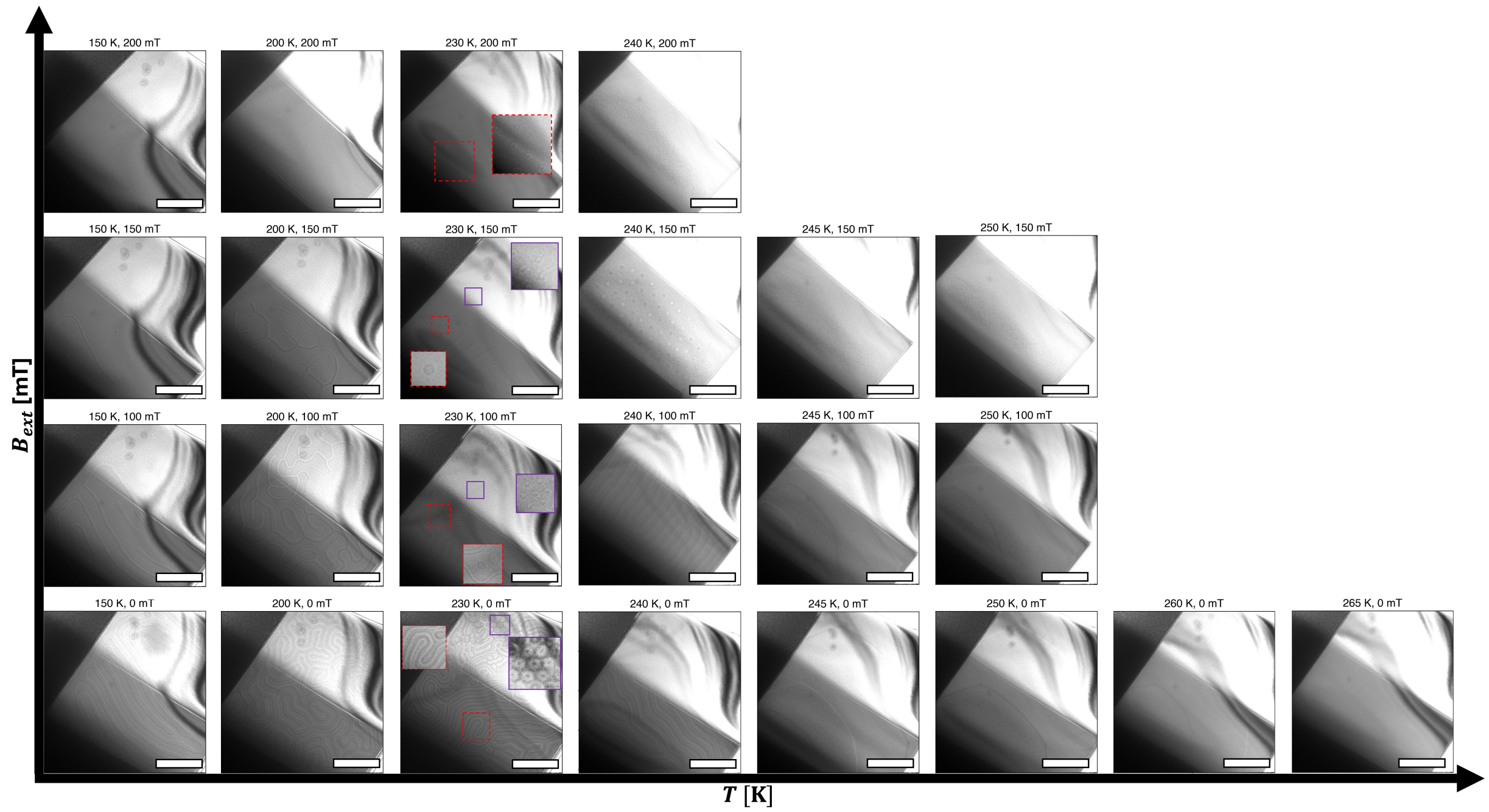

FIG. S5 : Selected LTEM micrographs tabulated to illustrate the magnetic state diagram. Images placed farther up the vertical axis correspond to elevated applied magnetic fields B_{ext} whereas the horizontal axis corresponds to increasing temperature. The micrographs chosen span a temperature range of $T = 150$ K to $T = 265$ K and magnetic field range of $B_{\text{ext}} = 0$ mT to $B_{\text{ext}} = 200$ mT. Scale bars are 1 μm.

respectively. This results in a scaling factor of $D_{91\ \text{nm}}/D_{43\ \text{nm}} = 1.46$, which matches the scaling value calculated using the EELS thickness measurements quite well ($\sqrt{d_{thick}/d_{thin}} = \sqrt{91/43} = 1.45$).

S6. MAGNETIC BUBBLE REVERIFICATION

Magnetic bubble formation was re-verified on a second set of crystals from a new growth batch than those used for the LTEM and DPC-STEM presented in the main manuscript. A lamella of $TmMn_6Sn_{4.2}Ga_{1.8}$ (displayed in Fig. S7(A)) is prepared from bulk single crystals using a focused ion beam (FIB) system (Helios DualBeam, Thermo Fisher Scientific) with Ga^+ ions. The lamella was oriented along the crystallographic [001] axis, as confirmed by selected area electron diffraction (SAED), shown in Fig. S7(B). Final thinning and surface

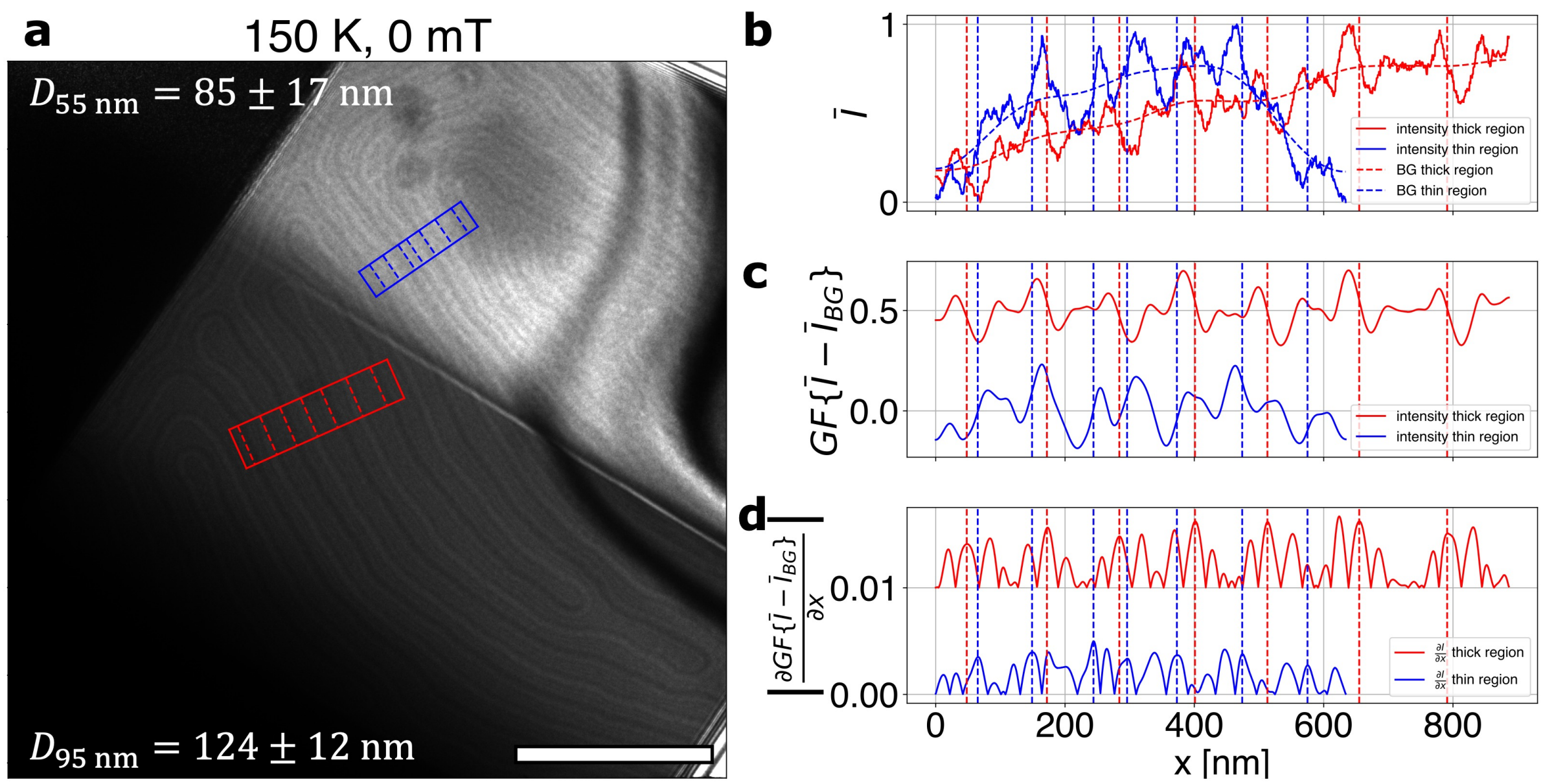

FIG. S6 : Measurement and comparison of characteristic magnetic domain sizes in different thickness regions at T=150 K and zero field. (**A**) LTEM micrograph of the $TmMn_6Sn_{4.2}Ga_{1.8}$ sample with stripey 180° magnetic domains present in both the thin (lighter) and thick areas of the lamella. Scale bar is 1μm. (**B-D**) The (**B**) average intensity $\overline{I}$ of 50 adjacent one-dimensional slices from the thick and thin regions of the sample indicated in (**A**) by red and blue rectangles, respectively. The low-frequency background intensity $\overline{I}_{\mathrm{BG}}$, which we extract using a gaussian filter with $\sigma_{\mathrm{GF}} = 50$ nm is plotted by dashed lines. (**C**) Plot of the gaussian-filtered, background-subtracted average intensity $GF\{\overline{I} - \overline{I}_{\mathrm{BG}}\}$ with $\sigma_{\overline{I}} = 7$ nm. (**D**) The absolute valued derivative of $GF\{\overline{I} - \overline{I}_{\mathrm{BG}}\}$ with respect to x. The seven domain wall positions are calculated as the x location with the largest valued derivative within a 40nm window of domain wall locations predetermined by eye. These domain wall x positions for the thick and thin regions are marked with red and blue dashed vertical lines, respectively in (**B-D**).

cleaning were performed at reduced ion energies of 5 kV and 2 kV to minimize surface damage and amorphization caused by ion milling.

Magnetic imaging was carried out using transmission electron microscopy in Lorentz mode (Spectra 300, Thermo Fisher Scientific) at the Notre Dame Integrated Imaging Facility (NDIIF). A double-tilt liquid nitrogen cooling holder (Gatan 636) was employed to

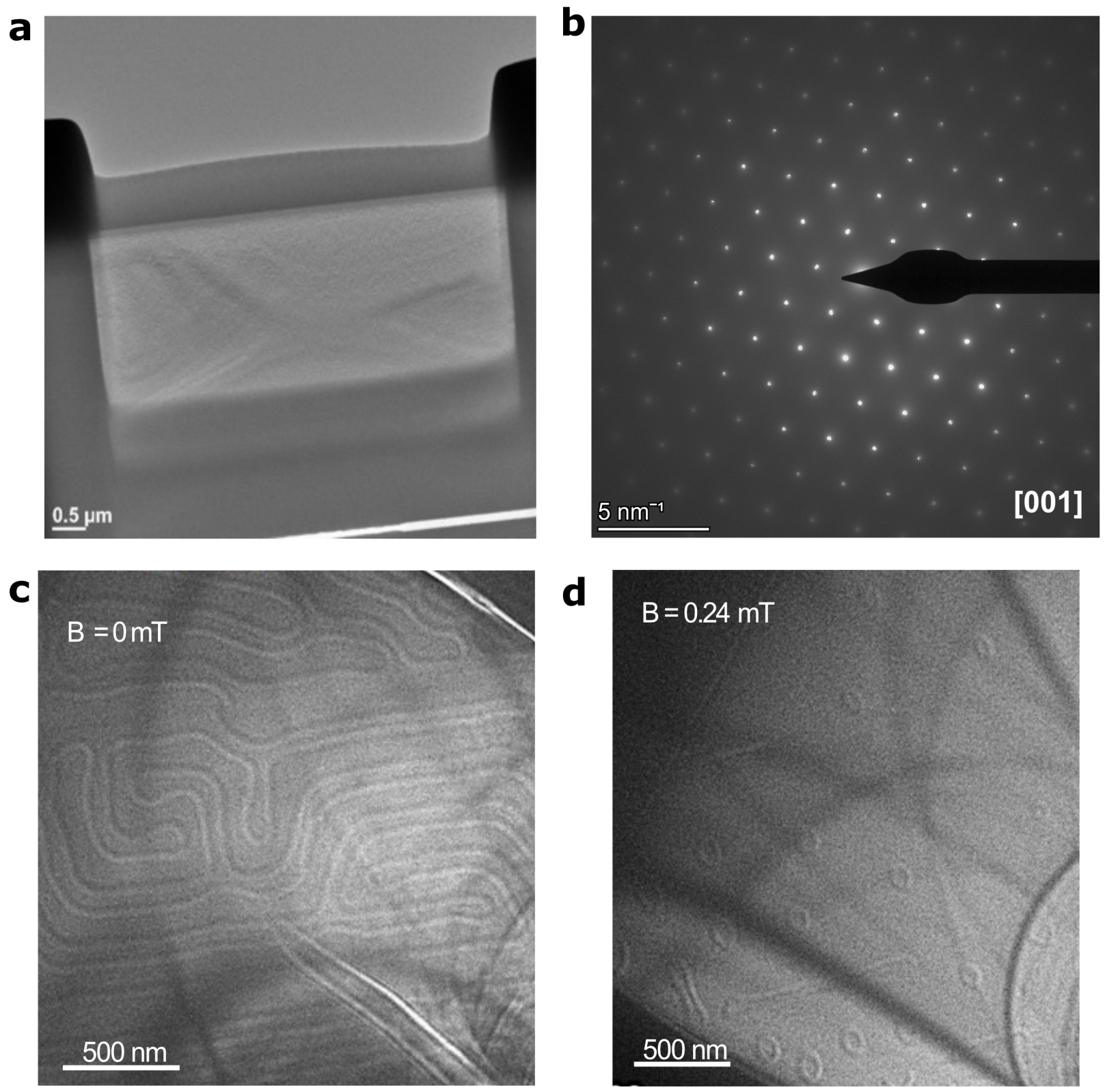

FIG. S7 : Reproducibility of magnetic bubbles in a second growth batch of $TmMn_6Sn_{4.2}Ga_{1.8}$. (**A**) SEM image of a FIB-prepared lamella oriented along the [001] direction, fabricated from a second independently grown $TmMn_6Sn_{4.2}Ga_{1.8}$ single crystal. (**B**) Selected area electron diffraction (SAED) pattern of the lamella, confirming orientation along the crystallographic *c*-axis. (**C**) Lorentz TEM image at 220 K and zero applied field reveals spontaneous stripe domains. (**D**) Magnetic bubbles emerge under an applied magnetic field of 0.24 mT.

enable low-temperature studies of magnetic domain structures. LTEM images were acquired across a range of temperatures, with magnetic field applied along the [001] crystallographic direction. Magnetic field strength was modulated from 0 T to 0.3 T by finely tuning the objective lens current. At 220 K, with 0 applied magnetic field, stripe domains (Fig. S7(C)) emerged spontaneously. Upon increasing the magnetic field, these stripe domains progressively fragmented into the bubbles (Fig. S7(D)).

S7. SUPPLEMENTARY MOVIE CAPTION

Movie S1. Helical switching of skyrmionic magnetic bubbles present in $TmMn_6Sn_{4.2}Ga_{1.8}$ while applying $B_{ext} = 125$ mT at $T = 210$ K with a beam current of 25.1 pA cm^{-2}. The bubbles analyzed in Figure 4 are circled and the interface between the $TmMn_6Sn_{4.2}Ga_{1.8}$ area and the Pt is marked by a solid blue line.

S8. SUPPLEMENTARY REFERENCES

---

[*] Equal contribution; melgazza@nd.edu

[†] Equal contribution; Corresponding Author; yasinfs@ornl.gov

[‡] Corresponding Author; nghimire@nd.edu

[1] J. Rodriguez-Carvajal, Physica B **192**, 55 (1993).

[2] M. Pajda, J. Kudrnovský, I. Turek, V. Drchal, and P. Bruno, Phys. Rev. B **64**, 174402 (2001), cond-mat/0007441.

[3] M. E. Gazzah, P.-H. Chang, Y. Lee, H. Bhandari, R. Regmi, X. Zhou, L. Ke, I. I. Mazin, and N. J. Ghimire, Phys. Rev. Mater. **9**, 114414 (2025).

[4] C. Kittel, Physical Review **70**, 965 (1946).
